# *Aloe vera*―An Extensive Review Focused on Recent Studies

**DOI:** 10.3390/foods13132155

**Published:** 2024-07-08

**Authors:** Alessia Catalano, Jessica Ceramella, Domenico Iacopetta, Maria Marra, Filomena Conforti, Francesca R. Lupi, Domenico Gabriele, Fernanda Borges, Maria Stefania Sinicropi

**Affiliations:** 1 Department of Pharmacy-Drug Sciences, University of Bari “Aldo Moro”, Via Orabona 4, 70126 Bari, Italy; alessia.catalano@uniba.it; 2 Department of Pharmacy, Health and Nutritional Sciences, University of Calabria, 87036 Rende, Italy; jessica.ceramella@unical.it (J.C.); mariamarra1997@gmail.com (M.M.); filomena.conforti@unical.it (F.C.); s.sinicropi@unical.it (M.S.S.); 3 Department of Information, Modeling, Electronics and System Engineering, (D.I.M.E.S.), University of Calabria, Via P. Bucci, Cubo 39C, CS, 87036 Rende, Italy; francesca.lupi@unical.it (F.R.L.); domenico.gabriele@unical.it (D.G.); 4 CIQUP-IMS—Centro de Investigação em Química da Universidade do Porto, Institute of Molecular Sciences, Department of Chemistry and Biochemistry, Faculty of Sciences, University of Porto, Rua do Campo Alegre s/n, 4169-007 Porto, Portugal; fborges@fc.up.pt

**Keywords:** *Aloe vera* L., biological activities, chemical composition, toxicological aspects

## Abstract

Since ancient times, *Aloe vera* L. (AV) has attracted scientific interest because of its multiple cosmetic and medicinal properties, attributable to compounds present in leaves and other parts of the plant. The collected literature data show that AV and its products have a beneficial influence on human health, both by topical and oral use, as juice or an extract. Several scientific studies demonstrated the numerous biological activities of AV, including, for instance, antiviral, antimicrobial, antitumor, and antifungal. Moreover, its important antidepressant activity in relation to several diseases, including skin disorders (psoriasis, acne, and so on) and prediabetes, is a growing field of research. This comprehensive review intends to present the most significant and recent studies regarding the plethora of AV’s biological activities and an in-depth analysis exploring the component/s responsible for them. Moreover, its morphology and chemical composition are described, along with some studies regarding the single components of AV available in commerce. Finally, valorization studies and a discussion about the metabolism and toxicological aspects of this “Wonder Plant” are reported.

## 1. Introduction

*Aloe vera* L. (AV), or *Aloe barbadensis* Miller or *Aloe barbadensis* Mill., is an interesting plant with diverse actions, belonging to the genus Aloe. A few years ago, it was considered to belong to the vast Liliaceae family, but in 1982, the botanist Reynolds placed it in the Aloaceae family [1]. It is a plant widely found in the hot and dry areas of the southern Mediterranean, the Middle East, North Africa (from Morocco to Egypt), Asia (especially in West India and Southwest Asia), Central America, Mexico, Cape Verde, Madeira, and the Canary Islands [2]. AV is broadly used in health and nutritional supplements in Chinese herbal medicine [3]. AV can develop in half-shadow sunlight or oblique sunlight; however, it requires almost 5–6 h of daylight [4]. AV has been defined “the Miracle plant” or the “Wonder plant” due to its amazing pharmacological activities, which are continuously studied [5,6], and Kumar et al. (2019) [7] recently indicated AV as “A miracle gift of nature”. In addition to its known overall beneficial effect on burn wound healing [8] and its use as laxative [9], AV has demonstrated various biological effects [10], such as anti-inflammatory [11], analgesic [12], immunomodulatory [13], anticonstipation, antioxidant [14], antiulcerogenic [15], anti-irritant, antimicrobial [16], anticancer [17], aphrodisiac [18], and antiviral, and has also shown usefulness against SARS-CoV-2 [19]. Moreover, its cardioprotective and antianginal [20] characteristics have been described, as well as its use in dentistry and oral health [21,22], as it is also used in mouthwashes [23,24]. Several reports refer to the usefulness of AV in polycystic ovary syndrome (PCOS), a multiple-endocrine disorder most frequently encountered in women of reproductive age associated with significant metabolic manifestations [25]. Recently, its usefulness in the prevention and treatment of chemotherapy-related or radiation-related oral mucositis has been addressed [26]. AV gel is also famous for its healing effects in the treatment of different ailments, especially gastrointestinal disorders [27,28]. Natural extracts of AV are able to re-equilibrate the gut microbiota and have also been suggested as potential candidates in the treatment of multiple sclerosis patients [29]. Several studies have also shown the positive effects of AV in skin disorders, including psoriasis [30], mouth sores, bedsores, dandruff, dry skin, cold sores, mouth ulcers [31], diseases related to hyperpigmentation (melasma, age spots, lentigines) [32], and as a protective agent against UV exposure [33]. In recent years, much information has also emerged on the use of this plant in combination with other naturally derived products [34,35,36,37]. Recently, the preparation of AV gel integrated with avobenzone, *N*-acetylcysteine, and glutathione has been suggested as an eco-friendly natural sunscreen gel with a low cost that provides effective photoprotection against UV-A radiation/sunlight [38]. Therapeutically, AV is also used to prevent progressive skin ischemia due to burns, frostbite, electrical injury, and intra-arterial drug abuse. Recent studies have addressed the evaluation of the potential of AV gel and whole leaf extract as drug permeation enhancers, in the delivery of protein and peptide drugs as well as other poorly bioavailable drugs via the oral, nasal, transdermal, and pulmonary routes of administration [39]. AV enhanced nasal drug delivery in both in vitro and ex vivo nasal epithelial models [40]. Kirby-Smith et al. (2023) [41] have recently reported a study on AV polysaccharides in microparticle formulations, which significantly improved nasal insulin delivery, used for the treatment of Alzheimer’s disease (AD) and diabetes mellitus (DM). Furthermore, AV is widely used to produce cosmetics, skin care products, and nutraceuticals. In cosmetics, it is used for the preparation of creams, lotions, soaps, and shampoos, while in the food industry, it is used for the preparation of health drinks [42], such as drinking yogurt [43], and to make curd, lassi, ice creams, and other desserts [1]. Finally, the use of AV as a food preservative has also been widely described for the prevention of postharvest losses, improving the shelf life of fresh fruits and vegetables [44,45], as well as as a sustainable green alternative to antibiotics in modern poultry production [46]. Several studies have addressed the preparation of nanoparticles containing AV, which usually increases its activities [47]. Furthermore, the addition of AV to chitosan has shown interesting results in diverse fields, by improving the activity and/or quality of chitosan [48,49,50]. Given its multiple activities, recent studies have often addressed the use of biofertilizers that could improve the growth and development of the AV plant [51]. Regarding the potential toxicity of AV, the topical use of gel is considered safe [52], whereas the safety of AV taken orally is still controversial. Some studies on AV extract or juice consider it to be safe [18,53], whereas other studies assess it to be toxic and to have adverse clinical effects [54,55,56]. During pregnancy or lactation, topical use is safe, with no known pregnancy-related adverse effects, whereas oral administration is generally not recommended; indeed, the strong laxative potential may cause stimulation of the uterus, contraction, and abortion [57]. The use of AV must also be avoided in people suffering from abdominal pain, appendicitis, or intestinal obstruction. Recently, the photocatalytic activity of AV zinc oxide nanoparticles, prepared with a green synthesis, has been described. They may reduce environmental pollution due to dye and drug releases from industries [58]. In this review, we summarize the pharmacological activities of AV and the main compounds responsible for its various activities. Literature research was done on the PubMed/MEDLINE, Scopus, and Google Scholar search engines using general keywords such as “*Aloe vera*”, “*Aloe barbadensis* Miller”, “*Aloe barbadensis* Mill.”, and “activities of *Aloe vera*”. All abstracts and full-text articles were examined for their relevance to this review. Diverse active compounds have been related to the biological activities of the plant, although it is likely that the synergism between different compounds is more likely the most important factor behind them [59].

## 2. *Aloe vera* Mill

**Figure 1:**
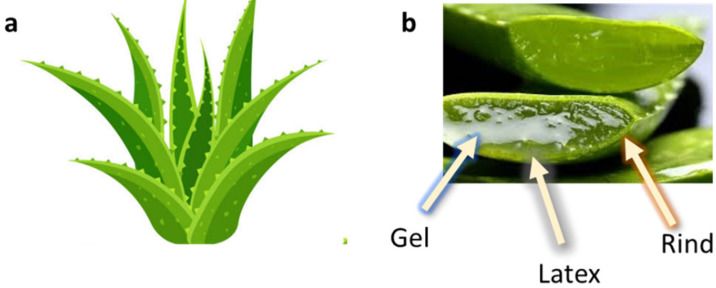
*Aloe vera* plant (**a**) and leaf (**b**).

The first modern reference to AV is found in 1655, from a translation of Dioscorides’ medical treatise *De Materia Medica* by John Goodyew [60]. Its name comes from the Arabic word “Alloeh”, which means “shining bitter substance” or “sparkling bitter material”, and the Latin word “vera”, which means “truth” [61,62]. In ancient times, it was used by the Egyptians as a component for embalming; indeed, they call it “the plant of immortality”, it was used as a healing agent but also for body care and hygiene. More than 2000 years ago, the Greeks regarded AV as “the universal panacea”. There are more than 350 species of *Aloe*, of which AV and *Aloe arborescens* are the most common. AV is a succulent species, native to South and East Africa, but thanks to its highly adaptable nature and popularity, it is now largely cultivated in many tropical and subtropical parts of the world and in Mediterranean regions. It is a monocotyledonous, perennial, succulent, herbaceous medicinal plant. AV occurs as a shrubby, perennial, green-colored plant with an average height of about 30 to 70 cm (Figure 1). At full maturity, the plant reaches a size characterized by green fleshy leaves 60–80 cm long with serrated edges (color varies from bright green to gray), with a basal width of about 8–10 cm [63]. It has yellow tubular flowers 25–35 cm in length set in a lean, loose spike, and fruits that contain many seeds, and can reach one meter in height. The leaf is the most important part because it contains the biological active ingredients [64]. Each leaf consists of three layers: the outer layer, called the peel or rind, which consists of 15–20 types of cells, which helps in the synthesis of carbohydrates and proteins and has a protective function; the middle layer, which contains the latex, a bitter yellow sap that includes anthraquinones and glycosides; inside, the inner layer is composed of the gel, which is transparent and contains 99% water, while the rest is represented by glucomannans, amino acids, lipids, chromones, sterols, and vitamins [65]. For centuries, AV gel has been used for curative and therapeutic purposes, and more than 75 nutrients, as well as 200 biologically active constituents, have been discovered within the gel, including hydrosoluble and liposoluble vitamins (vitamins A, C, E, and B12), enzymes (amylase, catalase, and peroxidase), minerals (zinc, copper, selenium, and calcium), and sugars (monosaccharides such as mannose-6-phosphate and polysaccharides such as glucomannans, including acemannan). Moreover, AV contains anthraquinones (such as aloin, emodin, and aloe-emodin), fatty acids, hormones (auxins and gibberellins), and more (salicylic acid, lignin, and saponins) [66,67]. Volatile compounds and ascorbic acid are present in the flowers, while polysaccharides, lignin, pectin, hemicellulose, and cellulose are present in the rind. 

## 3. Pharmacological Activities

The implications of AV, as the whole leaf extract, the gel, and the latex, for health maintenance through the modulation of various biological activities have been widely described worldwide [68,69]. The most common ones and recent studies are summarized below (Table 1). Particularly, surveys regarding the extracts obtained from the plant and single components extracted from AV are reported, as well as studies on commercially available single components of AV.

### 3.1. Aloe vera Anti-Inflammatory Activity

Tornero-Martínez et al. (2022) [70] studied AV and its fermented extracts for their anti-inflammatory effect on human glioblastoma/astrocytoma cell line U373 MG, which develops a neuro-inflammatory profile. When assessing the status of pERK, both AV gel and its extracts showed an anti-inflammatory effect comparable to or higher than that of diclofenac, used as a control, showing a strong inhibitory effect on NF-kB (nuclear factor κB) phosphorylation. Paul et al. (2021) [71] studied the effects of AV gel homogenate in vitro in preventing membrane lysis and protein denaturation. Moreover, the regulation of inflammation-mediator gene expression (TNF-α and COX-2) was studied in vivo in Freund’s complete adjuvant-induced inflammatory arthritic Wistar albino rats. It was found that AV gel homogenate determined the lysosomal membrane stabilization and downregulation of TNF-α and COX-2 gene expression, thus showing anti-inflammatory activity. Babu & Noor (2019) [72] studied the anti-inflammatory activity of the peptide/polypeptide fraction of AV, obtained through trichloroacetic acid precipitation. In this study, homogenized AV gel was found to inhibit heat-induced protein denaturation and stabilize the membranes of red blood cells in a dose-dependent manner. Its anti-inflammatory potential was confirmed under inflammatory conditions in vivo using a rat paw edema model, through the injection of Freund’s adjuvant in the hind paw, and then the measurement of the levels of aspartate transaminase (AST) and alanine aminotransferase (ALT) in plasma. Rauwald et al. (2021) [73] demonstrated the importance of low molecular phenolic polyketides for the anti-inflammatory activity of AV preparations, isolating new compounds from AV that were active in the 5-lipoxygenase (5-LOX) test. Other studies related to the anti-inflammatory activity of AV in skin diseases and gastrointestinal diseases are detailed below. The anti-inflammatory activity of AV has been also related to its anti-bradykinin activity, due to the enzyme bradykinase, which reduces inflammation [74,75].

### 3.2. Aloe vera Antioxidant Activity

The antioxidant activity of AV extracts has been described in recent decades [76]. Nejatzadeh-Barandozi et al. (2013) [77] studied a 95% ethanol AV lyophilized leaf gel extract that showed antioxidant activity, as assessed by oxygen radical absorbance capacity (ORAC) and Ferric Reducing Ability of Plasma (FRAP) assays. The authors suggest that the antioxidant activity is related to polyphenols, indoles, and alkaloids. Pop et al. (2022) [78] assess that the antioxidant activity is related to anthraquinones, chromones, and AV polysaccharides (APSs), as detailed below. Manye et al. (2023) [3] reported a recent study on aqueous and methanol extracts obtained using the maceration technique applied to AV Burman leaves. The reducing power activity and 1,1-diphenyl-2-picrylhydrazyl (DPPH) free radical scavenging activity were evaluated. The methanol extract produced a better reducing power activity (EC_50_ = 249,316 µg/mL), whereas the aqueous extract was more active in the DPPH activity (IC_50_ = 54.0 µg/mL). Quercetin was used as a standard (EC_50_ = 25,325 µg/mL and IC_50_ = 36.8 µg/mL, respectively). Both the extracts revealed the presence of phenols, tannins, and flavonoids. Other studies are detailed below concerning antioxidant activity with radioprotective effects and the antibacterial activity of AV and studies on skin disorders.

### 3.3. Aloe vera Antibacterial Activity

The antibacterial activity of AV has been documented against Gram-positive and Gram-negative bacteria [79]. Lawrence et al. (2009) [80] reported a study on ethanol, methanol, and acetone extracts of AV gel against four Gram-positive (*Staphylococcus aureus*, *Streptococcus pyogenes*, *Bacillus subtilis*, *Bacillus cereus*) and four Gram-negative bacteria (*Escherichia coli*, *Pseudomonas aeruginosa*, *Salmonella typhi* and *Klebsiella pneumoniae*) using the agar well diffusion method. The ethanol and methanol extracts showed antibacterial activity, whereas the acetone extract demonstrated low or no activity. The authors identified *p*-coumaric acid, ascorbic acid, pyrocatechol, and cinnamic acid as compounds responsible for the antimicrobial activity. In the study by Nejatzadeh-Barandozi et al. (2013) [77], antibacterial activity against *S. aureus*, *S. pyogenes*, *P. aeruginosa*, and *E. coli* was reported for three different extracts of AV gel, specifically aqueous, ethanol, and acetone extracts. The maximum antibacterial activities were observed for the acetone extract. The antibacterial activity was higher against *S. pyogenes* and *P. aeruginosa* in comparison to *S. aureus* and *E. coli*. An important antibacterial activity of AV concerns the treatment of peptic ulcers [81], thanks to its activity against the Gram-negative *H. pylori*, a common bacterium affecting 50% of people worldwide [82]. The incorporation of AV gel with chitosan nanoparticles has shown enhanced bacteriostatic activity with respect to AV gel against *H. pylori*, as well as antioxidant and hemolysis inhibition [83]. The use of AV in tuberculosis has been reported by Azal et al. (2023) [84], who demonstrated that AV extract in vitro was effective in inhibiting the growth of *Mycobacterium tuberculosis* H37Rv strains, used in combination with second-line antitubercular drugs (ethionamide (ETH) or *para*-aminosalicylic acid (PAS)) and independently. Mawarti et al. (2022) [85] reported that AV ethanol extract was active against *M. tuberculosis* H37Rv and MDR TB strains HE (resistant to isoniazid and ethambutol) and SR (resistant to streptomycin and rifampicin) at a concentration of 50 mg/mL.

Additionally, the antimicrobial effectiveness of AV against *Enterococcus faecalis* [86], a bacterium responsible for oral cavity infections [87], is currently under consideration. The study by Arsene et al. (2022) [88] was carried out on AV extract against selected resistant Gram-negative bacteria involved in urinary tract infections (UTIs), namely, *Achromobacter xylosoxidans* 4892, *Citrobacter freundii* 426, *E coli* 1449, *Klebsiella oxytoca* 3003, *Moraxella catarrhalis* 4222, *Morganella morganii* 1543, *P. aeruginosa* 3057, and a reference strain *E. coli* ATCC 25922. AV demonstrated interesting antibacterial activity, with MIC and MBC values ranging from 0.625 to 5 mg/mL and 5 to 10 mg/mL, respectively, against all the strains tested, with the exception of *E. coli* 1449, which was totally non-susceptible. AV also demonstrated dose-dependent antibacterial effects, and the reference strain *E. coli* ATCC 25922 was the most susceptible, with MIC = 0.625 and IZ = 19 mm at 20 mg/mL. AV extract also showed antibiofilm activity, which was strong at twice the MIC value (93–100% of biofilm formation inhibition), moderate at half of the MIC value (32–41%), weak at one-quarter of the MIC value (14–21%), and nil at an eighth of the MIC value. Recent studies report the evaluation of nanoparticles containing NiO [89] and silver [90] for their antibacterial activity, even against multidrug-resistant microorganisms. Kong et al. (2017) [91] described a new naphthalene derivative, namely 3-hydroxy-1-(1,7-dihydroxy-3,6-dimethoxynaphthalen-2-yl)-propan-1-one, from AV displaying prominent antibacterial activity against methicillin-resistant *S. aureus* (MRSA) strain (MIC_90_ = 48 ± 4 mg/L), even stronger than that of levofloxacin (MIC_90_ = 58 ± 5 mg/L), used as positive control. Other studies of its antibacterial effects are reported below, in the studies regarding the use of AV in dentistry and the preservation of fruits and vegetables. Recently, Alghamdi et al. (2023) [92] studied the antimicrobial activities of an ethanolic and an aqueous extract of AV collected from the Jeddah, Al Baha, and Taif areas of the Kingdom of Saudi Arabia, along with an extract of *Opuntia-ficus indica* [93]. The aqueous extract showed activity against fungi, specifically *Aspergillus chevalieri* (IZD = 0.33 ± 0.12 mm), *Talaromyces funiculosus* (0.27 ± 0.06 mm), and *Penicillium funiculosum* (0.57 ± 0.40 mm) that was higher than that of the standard itraconazole (0.27 ± 0.12 mm; 0.13 ± 0.06 mm, and 0.23 ± 0.15 mm, respectively), and antibacterial activity against *Shigella* sp. (IZD = 0.47 ± 0.25 mm) that was higher than that of ciprofloxacin (IZD = 0.23 ± 0.06 mm). The ethanolic extract showed high activity against *A. chevalieri* (IZD = 1.00 ± 0.50 mm) compared to itraconazole, and also activity towards *T. funiculosus* (0.13 ± 0.12 mm) and *P. funiculosum* (0.20 ± 0.10 mm) that was similar to that of itraconazole, and residual antibacterial activity against *Shigella* sp. (IZD = 0.17 ± 0.06 mm), even though it was lower than that of the reference. 

**Table 1:** Studies of anti-inflammatory, antioxidant, and antibacterial effects of AV gel.

Activity			Ref.
Anti-inflammatory activity			[70]
	In vitro against human glioblastoma/astrocytoma cells (U373 MG)	NF-κB phosphorylation inhibition	
	In vivo on Wistar albino rats	Lysosomal membrane stabilization; downregulation of TNF-α and COX-2 gene expression	[71]
	In vitro evaluation of protein denaturation and stabilization of RBC membrane In vivo by rat paw edema model	In vitro: IC_50_ = 218.9 ± 15.6 μg/mL (protein denaturation); IC_50_ = 275.9 ± 19.1 μg/mL (stabilization of RBC membrane) In vivo: AST and ALT levels decreased by around 44.5% and 41.8%, respectively	[72]
Antioxidant activity			
	In vitro reducing power and DPPH radical scavenging activity	EC_50_ = 249,316 µg/mL: reducing power activity (methanol extract); IC_50_ = 54.0 µg/mL in DPPH assay (aqueous extract)	[3]
Antimicrobial activity			
	In vitro activity against Gram-positive and Gram-negative bacteria	Ethanol extract: IZD = 23.33 mm (*B. cereus*) IZD = 22.33 mm (*S. pyogenes*) IZD = 23.00 mm (*P. aeruginosa*) IZD = 22.66 mm (*K. pneumoniae*) Methanol extract: IZD = 22.33 mm (*B. cereus*) IZD = 15.00 mm (*S. pyogenes*) IZD = 10.66 mm (*P. aeruginosa*) IZD = 14.00 mm (*K. pneumoniae*)	[80]
	In vitro studies against *M. tuberculosis* H37Rv strains (ATCC 27294)	AV = 50 μg/mL ETH = 6.25 μg/mL AV + ETH = 12.5 μg/mL PAS: 3.12 μg/mL = AV + PAS: 25 μg/mL AV + ETH + PAS = 12.5 μg/mL	[84]
	In vitro studies against MRSA	3-hydroxy-1-(1,7-dihydroxy-3,6-dimethoxynaphthalen-2-yl)-propan-1-one obtained from AV: MIC_90_ = 48 ± 4 mg/L	[91]
	In vitro studies against fungi and bacteria	*Aqueous extract* Fungi: IZD = 0.33 ± 0.12 mm (*A. chevalieri*) IZD = 0.27 ± 0.06 mm (*T. funiculosus*) IZD = 0.57 ± 0.40 mm (*P. funiculosum*) Bacteria: IZD = 0.47 ± 0.25 mm (*Shigella* sp.) *Ethanolic extract* Fungi: IZD = 1.00 ± 0.50 mm (*A. chevalieri*) IZD = 0.13 ± 0.12 mm (*T. funiculosus*) IZD = 0.20 ± 0.10 mm (*P. funiculosum*) Bacteria: IZD = 0.17 ± 0.06 mm (*Shigella* sp.)	[92]

Abbreviations: NF-κB: nuclear factor κB; TNF-α: tumor necrosis factor-α; COX-2: cyclo-oxygenase-2; IC_50_: half-maximal (50%) inhibitory concentration; AST: aspartate transaminase; ALT: alanine aminotransferase; DPPH: 1,1-diphenyl-2-picrylhydrazyl; IZD: inhibitory zone diameter; ETH: ethionamide; PAS: *para*-aminosalicylic acid; MRSA: methicillin-resistant *S. aureus* strain; MIC: minimum inhibitory concentration.

### 3.4. Aloe vera Antifungal Activity

AV gel is considered a natural fungicide [94]. In the last decade, studies on AV leaf gels showed its fungistatic activity against *Candida* spp. Das et al. (2011) [95] reported a study on a protein of 14 kDa isolated from the AV leaf gel that showed activity against *Candida albicans*, *Candida parapsilosis*, and *Candida krusei*. Bernardes et al. (2012) [96] also demonstrated the antifungal activity of AV extract against *C. albicans*. The authors found that AV fresh leaf plant extract can inhibit both the growth and the formation of the germ tube by *C. albicans*, which is essential for virulence. Thaweboon et al. (2020) [97] recently studied an AV toothpaste containing 10% aqueous extract of the AV leaf, developed by the Thailand Institute of Scientific and Technological Research, demonstrating its antifungal activity against *C. albicans*. Several other studies on antifungal activity are described below regarding AV’s immunomodulatory activity, as well as oral cavity disorder studies. Añibarro-Ortega et al. (2019) [98] studied extracts from AV leaves (fillet, mucilage, and rind) and flowers for their antifungal activity. The fillet, rind, and flower extracts showed an interesting antifungal activity against *Aspergillus flavus*, *Aspergillus niger*, *Penicillium funiculosum*, and *C. albicans* (clinical isolate Ibis 475/15), which was higher than that of ketoconazole. A successive study by the same group [99] showed that aloesin-rich extracts, obtained from the rind, showed antioxidant activity, by inhibiting the formation of TBARS, and antimicrobial effects, mainly against *S. enterica* serovar Typhimurium, *Listeria monocytogenes,* and *C. albicans*.

### 3.5. Aloe vera Antiparasitic Activity

Dinesh et al. (2015) [100] described a study concerning the use of AV leaf extract and silver nanoparticles as effective candidates against mosquitos and bacteria. Mosquitocidal properties were assessed in a laboratory against the larvae (I-IV instar) and pupae of *Anopheles stephensi*, the mosquito that represents one of the most important malaria vectors in India and other West Asian countries. Good results were observed, with AV-synthesized silver nanoparticles leading to *A. stephensi* larval reductions of 74.5, 86.6, and 97.7%, after 24, 48, and 72 h, respectively. Moreover, the antiplasmodial potential of AV and *Allium sativum* on *Plasmodium* berghei-infected mice was reported by Adebayo et al. (2018) [101]. Zanzarin is a lotion based on coconut oil (*Cocos nucifera)*, jojoba oil (*Simmondsia chinesis*), and AV (Engelhard Arzneimittel GmbH & Co. KG, Niederdorfelden, Germany), sold as a cosmetic with repellent activities against ticks and biting insects. Its effectiveness in preventing infestation with *Tunga penetrans,* responsible for tungiasis [102], an important but highly neglected cause of morbidity in resource-poor communities in sub-Saharan Africa and South America, and sand flea disease, has been demonstrated [103,104]. The therapeutic potential of AV in leishmaniasis has been reported [105]. The leaf exudate of AV upon oral or subcutaneous administration to *Leishmania donovani*-infected BALB/c mice at a dose of 15 mg/kg body weight for 5 days reduced the parasite burden by >90% in the liver, spleen, and bone marrow [106]. 

### 3.6. Aloe vera Antiviral Activity

AV has been widely studied for its antiviral activity [107,108], as summarized in Table 2. One of the earliest reports on the antiviral activity of AV leaf extracts regarded human cytomegalovirus (HCMV), studied by plaque inhibition tests, flow cytometry, and morphometry assays [109]. In this study, the application of the extracts to HCMV-infected cells, in the middle stages of the viral infection cycle, determined a reduction in the HCMV plaque formation, suggesting that the extracts inhibited HCMV DNA synthesis. Choi et al. (2019) [110] demonstrated the usefulness of AV ethanol extract in inhibiting the replication of influenza A virus in Madin–Darby canine kidney (MDCK) cells and inhibited autophagy induced by influenza A virus in MDCK cells. Moreover, post-treatment with AV ethanol extract limited the viral matrix protein 1 (M1), matrix protein 2 (M2), and hemagglutinin (HA) mRNA synthesis and inhibited their expressions. Flavonoids (quercetin, catechin hydrate, and kaempferol) were identified as the active compounds. Gansukh et al. (2018) [111] reported the study of an AV aqueous extract, which showed excellent anti-influenza A activity in MDCK cells, comparable to that seen in the methanolic extracts, and no cytotoxicity, thus overcoming the limit of the high cytotoxicity of the methanolic extract. Rezazadeh et al. (2016) [112] described the anti-herpes simplex virus type 1 (HSV-1) activity exerted in vitro by AV gel at different concentrations, assessed by plaque reduction assays in Vero cells infected with HSV-1. The highest anti-HSV-1 antiviral activity was observed for AV gel at a 5% concentration, while 1, 0.5, and 0.2% concentrations had significantly lower activity. There was no significant toxic effect for the concentrations ranging from 0.2 to 5%. The authors also suggested AV gel as a useful topical treatment for oral HSV-1 infections. An in vitro study on APSs extracted from AV leaves, through water extraction and ethanol precipitation, was reported by Sun et al. (2018) [113], revealing that APS could inhibit the replication of a H1N1 subtype influenza virus, directly interacting with influenza virus particles. Experiments on PR8 (H1N1) virus infection in mice demonstrated that APS considerably ameliorated the clinical symptoms and the lung damage of the infected mice, and significantly reduced the virus loads and mortality. Ng et al. (2017) [114] also reported a study on the activity of AV crude extract against murine norovirus 1 (MNV1), showing dose-dependent inhibitory effects on MNV1. AV applied to fresh food surfaces was also able to reduce MNV1 infectivity both on the food vegetable surface and in liquid media, thus suggesting its use for treating food and preventing foodborne viral infections. The antiviral properties of AV are also helpful for the treatment of cold sores (*Herpes simplex*) and shingles (*Herpes zoster*) [60]. Moreover, AV contains zinc, which can inhibit the replication of retroviruses, including SARS-CoV-1 [115]. In the aftermath of the COVID-19 pandemic [116], scientific interest in the search for compounds with antiviral activity has increased. At present, there is no effective treatment against COVID-19 and post-COVID, even though some drugs already on the market have been recommended [117,118]. Different studies suggested that AV could be used as an antiviral agent against SARS-CoV-2 [119,120,121,122,123,124]. The Unani formulation “Tiryāq-i-WabāI” contains Sibr (*Aloe vera* (L.) Burm. f.), Murr Makki (*Commiphora myrrha* (T.Nees) Engl.), and Zāfrān (*Crocus sativus* L.); it has long been used in cholera, plague, and other epidemic diseases, and it has been recently suggested as the most recommended prophylactic and curative drug during COVID-19 and future epidemics [125]. 

### 3.7. Aloe vera Immunomodulatory Activity

The immunomodulatory activity of AV has been recently reviewed [126]. Im et al. (2010) [127] studied the in vivo effects of the oral administration of processed AV gel, which significantly reduced the growth of *C. albicans* in the spleen and kidney after intravenous injection of *C. albicans*. It also reduced the growth of *C. albicans* in streptozotocin-induced diabetic mice and enhanced ovalbumin-specific cytotoxic T lymphocyte generation only in high-fat diet-induced diabetic mice, but not in normal ones. The in vivo study by Madan et al. (2008) [128] also evidenced that AV gel extract had immunostimulatory effects on Swiss albino mice of either sex (300 mg/kg, i.p). Low or no effects were observed at a 150 mg/kg dose. López et al. (2019) [129] studied the immunomodulatory effect on human macrophage-like THP-1 cells induced by AV gel commercial powders used as food supplements provided by the Mexican facilities of a US enterprise. The authors demonstrated that the results varied on the basis of the fiber and polysaccharide content. Mosayebi et al. (2009) [130] studied the immunomodulatory activity of AV in an animal model of multiple sclerosis, a neurological and inflammatory autoimmune disease of the central nervous system in which the selective activation of T and B lymphocytes prompts a reaction against myelin, inducing demyelination and axonal loss. Studies were carried out in vivo in mice on tumor necrosis factor-alpha (TNF-α), and the development of experimental autoimmune encephalomyelitis (EAE) was evaluated. The treatment with AV extract significantly reduced the clinical signs of experimental autoimmune encephalomyelitis and delayed the onset of the disease. Mononuclear cells isolated from the spleens of mice treated with AV showed a significant reduction in TNF-α, compared with the control group. Other studies regarding its immunomodulatory activity are described below, including dentistry and oral disorder studies. 

### 3.8. Aloe vera Anticancer Activity

A particularly interesting field of application is the use of AV as a chemo-preventive and anticancer agent [131]. Tong et al. (2021) [132] studied the anticancer activity of an AV extract, C (AVBEC). In breast (MCF-7 and MDA-MB-231 with different malignancies) and lung cancer (small cell lung cancer cell NCI-H 524 and non-small cell lung cancer cell NCI-H 1975) cells in vitro, AVBEC induced cancer cell apoptosis, likely due to the modulation of mitochondrial function. In vivo, it did not cause toxic changes in the main organs or the peripheral and central immune system in Sprague Dawley rats. Majumder et al. (2020) [133] reported in vitro and in silico studies on an AV leaf extract in breast cancer. The extract can effectively inhibit the proliferation of breast cancer MCF-7 cell lines without any cytotoxic effect on healthy noncancerous NIH-3T3 cells, compared with the standard drug, tamoxifen. Shalabi et al. (2015) [134] reported the antitumor properties of AV extract, which suppressed the growth of hepatocellular carcinoma (HepG2) cells and induced cytotoxic effects, activating the apoptotic pathways. Karpagam et al. (2019) [135] showed that AV ethanolic leaf extract showed cytotoxicity against HepG2, HeLa (human cervical carcinoma), and A549 (human lung adenocarcinoma epithelial) cell lines. This was primarily ascribed to anthraquinones and APSs, as detailed below. However, aloctin, a lectin purified from AV leaves, has been shown to significantly increase the cytotoxic effect of imatinib, in a dose-dependent manner, in human gastric AGS adenocarcinoma cells [136]. The use of AV combined with carbon-based nanomaterials has been recently suggested as an antineoplastic agent in the treatment and prevention of melanoma [137]. The use of AV in liver cancer or hepatocarcinoma (HCC) has been recently discussed [138]. Finally, the use of AV in diseases related to cancer is often reported. AV has been suggested for the prevention and treatment of chemotherapy-related and radiation-related oral mucositis [139], for chemotherapy-induced phlebitis [140], and for chemotherapy-induced hyperpigmentation [141].

### 3.9. Aloe vera Radioprotective Effects

The radioprotective effect of AV and other natural products has been described in the literature [142]. Kumar & Tiku (2015) [143] studied acemannan, extracted from fresh leaves of AV, for its radioprotective effects, as pre- and post-irradiation treatment, in whole-body irradiated (WBI) Swiss albino mice. AV increased the mice’s survival by protecting against radiation damage, upregulating the immune system, and inducing proliferation of the hematopoietic cells. Moreover, acemannan was shown to be nontoxic at high doses. Bala et al. (2017) [144] suggested the radioprotective activity of AV against X-ray-induced testicular dysfunction. The study was carried out using AV extract against whole-body X-ray irradiation-induced testicular alterations in male BALB/c mice. Results showed that the pre-treatment with AV extract of irradiated mice produced a better profile in terms of the antioxidant status, inhibition of lipid peroxides, apoptotic cell formation, and enhanced testicular parameters in comparison to the control group. Moreover, in vitro and in vivo studies regarding the radio-modulatory effects of AV on the hepatic and renal tissues of X-ray-irradiated mice showed that AV may serve to boost the antioxidant system, providing protection against hepatic and renal damage caused by X-rays [145]. Farid et al. (2022) [146] studied the effect of AV gel on enhancing the proliferation and differentiation of mesenchymal stem cells (MSCs), biologically active precursor cells that can self-renew and develop into diverse types of cells. Lyophilized AV gel was used together with bone marrow MSC transplantation against radiation-induced liver damage in X-ray-irradiated Sprague Dawley male rats, showing improvement in liver function and a decrease in fibrotic markers, oxidative stress, and pro-inflammatory cytokines. Moreover, a reduction in the pathological alterations in the rats’ livers and a reduced NF-κB activation were observed. Thus, AV gel was suggested for its potential in regenerative medicine.

### 3.10. Aloe vera and Hepatoprotection and Renoprotection 

It has been reported that AV has a protective effect in organs against induced hepatotoxicity and nephrotoxicity. Kim et al. (2009) [147] studied the hepatoprotective effects of ACTIValoe^®^N-931 complex, a mixture of AV and *Silybum marianum*, against acute and chronic carbon tetrachloride-induced liver injuries. In both acute hepatotoxicity and liver fibrosis, serum aminotransferase levels and lipid peroxidation were enhanced, and the hepatic glutathione content was reduced. The administration of ACTIValoe^®^N-931 complex prevented these effects. As recently summarized by Aladejana et al. (2023) [148], several polyherbal formulations containing AV have demonstrated hepatoprotective activities against D-galactosamine- and carbon tetrachloride-induced hepatotoxicity in mice. Gupta et al. (2019) [149] reported that the administration of AV leaf aqueous extract on Wistar rat liver was able to markedly protect it from pesticide-induced toxicity (cartap and malathion). Pesticides significantly induced oxidative stress, which was substantially reduced by the application of AV extract. Recently, Al-Abbassi et al. (2023) [150] showed that AV gel extract had hepatoprotective effects against azathioprine, with significant changes in levels of biochemical markers in vivo in adult albino rats. The hepatoprotective effect of AV may be related to glutathione (GSH)-mediated detoxification [151]. Other possible mechanisms of the hepatoprotective effects exerted by AV gel have been reviewed by Handayani et al. (2021) [152]. Most of them are related to its protective effect against inflammation and oxidative stress. Several compounds may have combination effects, or several targets lead to synergistic effects. It has also been reported that AV may prevent kidney disease or stones [153]. Virani et al. (2016) [154] reported that AV is able to attenuate gentamicin-induced nephrotoxicity in Wistar albino rats, and Iftikhar et al. (2015) [155] described the nephroprotective effect of the leaves of AV against diclofenac sodium-induced toxicity in albino rabbits. Chatterjee et al. (2012) [156] reported the protective effects of AV aqueous leaf extract on gentamicin- and cisplatin-induced nephrotoxicity in Wistar rats. The study by El-Shafie et al. (2015) [157] described the curative effect of orally consumed AV juice on ochratoxin A-induced nephrotoxicity in rats. Ahmed et al. (2022) [158] reported the organoprotective ability of AV gel ethanolic extracts against streptozotocin-induced pancreatic, renal, and hepatic toxicity in female albino Wistar rats, by using metformin as a positive protective control in vivo. The hepatoprotective and nephroprotective activities of AV were demonstrated. Finally, AV extracts may help to reduce hepatic and renal heavy metal-mediated toxicity in experimental animals [159].

### 3.11. Cardioprotective Effects of Aloe vera

The cardioprotective effect of AV is described in the literature [5]. Kaithwas et al. (2011) [160] found that AV gel produced dose-dependent protection against doxorubicin-induced cardiotoxicity and calcium overload in albino rats. Yang et al. (2017) [161] reported a study on a selenium-containing polysaccharide isolated from the crude polysaccharides of AV leaves. It demonstrated a cardioprotective effect, increasing glutathione peroxidase activity in cardiac tissues and decreasing the incidence of ischemia-reperfusion injury. 

### 3.12. Aloe vera and Skin Disorders

AV is widely studied in skin disorders, including skin moisturizing, wound healing and care, frostbite, and ischemic skin insults in adults [162,163,164] and children [165]. Scientific studies have shown that the AV gel can increase the flexibility and reduce the fragility of the skin, since 99% of the gel is made of water. Additionally, mucopolysaccharides, along with the amino acids and zinc present in AV, can improve skin integrity, leading to moisture retention and erythema reduction, thus helping to prevent ulcers. AV can be utilized in different conditions like erythema, genital herpes, seborrheic dermatitis, psoriasis, verbal lichen planus diseases, and UV-induced erythema [166]. In vivo skin hydration and anti-erythema effects of AV gel were demonstrated by Fox et al. (2014) [167]. AV is currently marketed for the treatment of hyperpigmentation and is included in cosmetic products [168]. AV gel has been demonstrated to inhibit the enzyme tyrosinase, a multifunctional catechol oxidase belonging to the type-3 copper-containing metalloenzymes, which is responsible for melanin production. Thus, AV’s use has been reported in diseases related to hyperpigmentation, melasma, age spots, and lentigines, caused by the overactivity of the tyrosinase enzyme [169]. 

Arbab et al. (2021) [170] studied AV extracts as antimicrobial agents against different bacterial isolates from skin infections in animals and concluded that ethanol extracts of AV, from both the leaf and root, are more efficacious than conventional extracts. Recent studies have addressed APS, which is the main bioactive component of AV [95], and is responsible for its action in the treatment of skin disorders. Studies in wound healing, acne vulgaris, alopecia areata, psoriasis, and frostbite are summarized in Table 3.

AV is able to promote skin wound healing without showing any toxic effects; thus, it is widely used as a therapy for burns [171,172]. Aulia & Pane (2022) [173] showed that AV extract could quicken the healing process of second-degree burn wounds in rats. A recent meta-analysis (2022) evidenced that topical AV usage for second-degree burn wound healing led to a significantly faster time to healing compared with other treatments [174]. Results in third-degree burns are often controversial. Takzaree et al. (2016) [175] studied the acceleration of wound healing after the topical application of AV gel in Wistar rats. Results showed that fibroblasts were significantly increased, and there was an increase in transforming growth factor-β (TGF-β) gene expression, ultimately accelerating the wound-healing process. Levin et al. (2022) [176] reported a systematic review and meta-analysis, comparing burn healing outcomes between silver sulfadiazine and AV in second- and third-degree burn wounds, based on six cohort studies and two randomized controlled trials in both animals and humans. Results show that time to healing benefitted from those burns on which AV was used. Imbarak et al. (2021) [177] demonstrated that the topical application of AV promoted burn wound healing, with wounds healing faster and better than with the intradermal injection of mesenchymal stem cells in experimentally induced deep second-degree burns in adult female albino rats. The study by Atiba et al. (2022) [178] considered both oral and topical applications of AV in deep second-degree burn wound healing in male Sprague Dawley rats. Beneficial effects were observed in both cases. Presumably, the mechanism of action is provided by boosting the growth factors and antioxidant status of skin tissue. An in vivo study carried out on 24 rats demonstrated that oral and topical AV formulations produce improved results in wound healing [179]. Kim et al. (2021) [180] studied the therapeutical potential of extracellular vesicles isolated from AV peels in wound healing by in vitro scratch assay using human keratinocytes (HaCaTs) and fibroblasts (HDFs). The expression of nuclear factor erythroid 2-related factor 2 (Nrf2) and its associated genes was also analyzed by quantitative RT-PCR. The extracellular vesicles isolated from AV peels could activate the antioxidant defense mechanisms and wound healing process via the Nrf2 activation, enhancing the migration ability of HaCaTs and HDFs and suggesting their usefulness for chronic wound treatment. Moreover, the use of AV-based hydrogels, which may incorporate various therapeutic agents, such as antimicrobial and anti-inflammatory agents, for wound dressing applications is widely described [181]. Moreover, AV gel has demonstrated interesting activity in diabetic foot ulcer (DFU) [182,183,184]. Finally, the incorporation of AV into chitosan dressings could potentialize the healing process by increasing the films’ stability at temperatures below 200 °C [185].

Acne vulgaris is one of the most prevalent skin diseases, affecting most adolescents and having a major impact on their quality of life and psychosocial well-being [186]. It presents both non-inflammatory and inflammatory skin lesions, the latter due to the bacterium *Propionibacterium acnes.* The effect of AV topical gel combined with tretinoin was studied in a randomized, double-blind, prospective trial in the treatment of acne vulgaris, showing that this combination was well tolerated and resulted in significantly greater improvement of mild to moderate acne vulgaris than that from tretinoin and placebo [187]. Recently, an anti-acne AV extract was tested in vitro against *P. acnes* by the agar diffusion method using several concentration variations: 2.5%, 5%, and 10%. Interesting results were obtained for the three extracts, with the 10% AV ethanol extract being the most active [188].

Alopecia areata is an ambiguous autoimmune disorder characterized by transient, nonscarring hair loss and preservation of the hair follicle, leading to progressive hair loss, usually in distinct nonscarring patches [189]. *P. acnes* and *Cutibacterium acnes* are the main causative bacterial agents of infection of the scalp in alopecia areata [190]. AV gel has been studied in alopecia areata since ancient times [191]. Hosny et al. (2022) [192] prepared a topical garlic oil containing finasteride-encapsulated nanotransferosomes (NTFSs). The formulation was added to the AV gel and used for the prevention of microbial growth at the scalp during the treatment of alopecia areata. The AV gel loaded with finasteride–garlic oil–nanotransferosomes provided sustained and targeted drug delivery with increased encapsulation efficiency, nontoxicity, non-irritancy, and significant inhibition of the microbial zone of the said bacterial colony and more effectively treated the alopecia areata. 

Psoriasis is considered a multifactorial and heterogeneous systemic disease with many underlying pathologic mechanisms [193]. AV shows an immunomodulatory effect, stimulating macrophages and lymphocytes to release NO and cytokines and activating the maturation of undeveloped dendritic cells [194]. Leng et al. (2018) [195] showed that APSs extracted from AV significantly diminished the TNF-α-stimulated elevation of HaCaT cell proliferation in a dose-dependent manner. Moreover, it increased the expression levels of inflammatory factors, including IL-8 and IL-12, in response to TNF-α. Thus, APS inhibited TNF-α-induced proliferation of keratinocytes and overactivation of the NF-κB signaling pathway. A recent review summarizing clinical and preclinical studies demonstrated that an ethanolic extract of AV has a positive effect on psoriatic lesions, similar to traditional drugs [196]. 

Frostbite is defined as the acute freezing of tissues when exposed to cold temperatures (below the freezing point of intact skin). The severity of the injury is related to the temperature gradient at the skin surface and the duration of exposure. Frostbite, once seen as a military problem, has become more prevalent among the civilian population over the past three decades [197]. It can lead to severe injury and bleeding complications, resulting in amputations [198]. AV is generally used for minimizing frostbite damage [199]. The topical use of AV wraps has been recently reported in a case of frostbite treated with delayed hyperbaric oxygen therapy [200]. ”Dermaide” *Aloe* cream is commonly applied on thawed tissue before dressing. It consists of AV gel and acts as a topical inhibitor of thromboxane. AV reverses progressive ischemia, as well as preserving the dermal microcirculation of cells, by preventing the systemic production and local effect of thromboxane [201].

**Table 3:** Preclinical and clinical studies on AV gel in skin disorders.

Activity			Ref.
Wound healing	In vivo studies in *Rattus norvegicus* Wistar strain rats	AV quickened the healing process in second-degree burn wounds	[173]
	In vivo acceleration of wound healing after topical application of AV gel in Wistar rats	AV increased fibroblasts and TGF-β gene expression	[175]
	Comparison of burn healing outcomes between silver sulfadiazine and AV in second- and third-degree burn wounds in both animals and humans	Time to healing benefitted those burns on which AV was used	[176]
	In vivo induced deep second-degree burns in adult female albino rats	Topical AV promoted burn wound healing, with wounds healing faster and better than with intradermal injection of mesenchymal stem cells	[177]
	In vivo studies in deep second-degree burn wound healing in male Sprague Dawley rats	Both oral and topical applications of AV demonstrated beneficial effects by boosting the growth factors and antioxidant status of skin tissue	[178]
	In vitro scratch assay in wound healing using HaCaTs and HDFs; the expression of Nrf2 and its associated genes was also analyzed by quantitative RT-PCR	The extracellular vesicles isolated from AV peels activated the antioxidant mechanisms and wound-healing process via Nrf2 activation and enhanced the migration ability of HaCaTs and HDFs	[180]
Acne vulgaris	Randomized, double-blind, prospective trial	Combination of topical AV and tretinoin greatly improved mild to moderate acne vulgaris with respect to tretinoin and placebo	[187]
	In vitro assay against *P. acnes* by the agar diffusion method	AV’s antibacterial activity against *P. acnes:* IZD = 8.8. mm (at 2.5% concentration) IZD = 9.8 mm (at 5% concentration) IZD = 12.9 mm (at 10% concentration)	[188]
Alopecia areata	In vitro drug release study for the prevention of microbial growth at the scalp during the treatment of alopecia areata	Topical AV gel loaded with finasteride–garlic oil–NTFS provided sustained and targeted drug delivery with increased encapsulation efficiency, nontoxicity, non-irritancy, and significant inhibition of the microbial zone of the said bacterial colony and more effectively treated the alopecia areata	[192]
Psoriasis	In vitro studies in human keratinocyte cell line HaCaTs using the CCK-8 assay; ELISA and Western blotting used to study the abundance of IL-8 and IL-12 in TNF-α-incubated culture medium and APS-treated HaCaT cells, respectively	APS significantly diminished TNF-α-stimulated HaCaT cell proliferation dose-dependently; it also increased the expression levels of IL-8-12 in response to TNF-α	[195]
Frostbite	In vitro and in vivo experiments and clinical trials using AV preparations	“Dermaide” *Aloe* cream (AV gel) is a topical inhibitor of thromboxane and reverses progressive ischemia and preserves the dermal microcirculation of cells	[201]

Abbreviations: TGF-β: transforming growth factor-β (TGF-β); Nrf2: nuclear factor erythroid 2-related factor 2; HDF: human dermal fibroblast; IZD: inhibitory zone diameter; NTFS: nanotransferosomes; APS: AV polysaccharide; TNF-α: tumor necrosis factor-α.

### 3.13. Aloe vera in Gastrointestinal Disorders

AV is known for its laxative properties and is used in adults and children [202,203]. Anthraquinones are the main compounds responsible for this activity [204]. *Lu-Hui*, the dried leaf juice extract of AV, is one of the most popular traditional Chinese medicines and is officially recorded in the *Chinese Pharmacopoeia* (*Pharmacopoeia of the People’s Republic of China*). It has been traditionally used for treating functional constipation [205]. The study by Yu et al. (2024) [204] suggests that aloin A, aloin B, and aloe-emodin, followed by aloeresin D, are the key constituents responsible for the anticonstipation activity. Gastroesophageal reflux disease (GERD) is a result of lower esophageal sphincter weakness, which returns the contents of the stomach to the esophagus. Its symptoms are represented by heartburn, food regurgitation, flatulence, belching, dysphagia, nausea, vomiting, and acid regurgitation. AV gel is known as a healing agent for the treatment of internal and external ailments (Table 4). Panahi et al. (2015) [206] reported the usefulness of AV syrup in GERD in a randomized clinical trial. It was found to be safe and well tolerated, reducing the frequencies of all the assessed GERD symptoms, with no side events requiring withdrawal. A recent review by Mahboubi (2021) [207] summarizes the studies regarding the effectiveness of AV gel in the management of GERD. It comprises five clinical studies in patients suffering from GERD treated with 10 mL AV gel syrup twice daily, in comparison to omeprazole, ranitidine, pantoprazole, or aluminum–magnesium hydroxide. It was found that AV gel syrup significantly eliminated the GERD symptoms without any adverse effects, when compared with omeprazole or ranitidine. Irritable bowel syndrome (IBS) is one of the most common functional gastrointestinal disorders globally, characterized by chronic abdominal pain or discomfort and changes in bowel frequency and/or pattern. A systematic review and meta-analysis of randomized controlled trials, non-randomized controlled trials, retrospective and prospective cohort studies, and controlled before-and-after studies was recently reported by Fong-Jaén et al. (2022) [208]. It evidenced that the consumption of AV improved health outcomes in adults with IBS. In addition to the above-described uses of AV in gastric ulcers and GERD, some AV products have shown therapeutic benefits in the symptomatic treatment of IBD [209,210], a very heterogeneous condition, historically subdivided into Crohn’s disease (CD) and ulcerative colitis (UC) [211]. Recently, Choi et al. (2023) [212] suggested the use of AV-derived nanovesicles as a safe treatment option for IBD. The therapeutic potential and molecular mechanisms of the nanovesicles were studied in a dextran sulfate sodium (DSS)-induced acute experimental colitis mouse model, and it was suggested that they were able to attenuate inflammation and enhance tight junction proteins for acute colitis treatment. Zhang et al. (2019) [213] demonstrated that glucomannan from an AV gel polysaccharide protected mice from DSS-induced colitis. The mechanism, recently discovered, resides in the ability of AV gel polysaccharide to maintain the intestinal barrier integrity by mitigating anoikis, through the Nrf2/mitochondria axis. Specifically, it reduced ROS levels by activating the Nrf2/Gpx2 cascade [214]. A recent study on subacute UC demonstrated that APSs extracted from AV were responsible for the anticolitic action, by alleviating colonic inflammation [215]. Ismaeil et al. (2020) [216] demonstrated that AV exerts an ameliorative effect on DSS-induced colitis in mice, functioning synergistically, with heat-killed *Lactobacillus plantarum* L.137 (HK L.137) being suggested as an effective strategy to prevent colitis. Studies on UC are often related to polysaccharides. In a placebo-controlled, randomized, double-blind trial involving 44 UC patients, reported by Langmead et al. (2004) [217], the effects of oral AV gel (200 mL/day) were studied over a period of four weeks. UC patients receiving oral AV gel showed a significant reduction in clinical disease activity scores (evaluated using the Simple Clinical Colitis Activity Index, SCCAI) and histological disease activity scores in comparison to the placebo group. The administration of AV gel did not determine any serious side effects. Babalola et al. (2022) [218] showed that treatment with AV gel attenuates acetic acid-induced UC in rats and significantly improves the clinical activity index and inflammation. Naini et al. (2021) [219] showed that AV extract exhibited a therapeutic effect in 2,4,6-trinitrobenzene sulfonic acid (TNBS)-induced colitis. The oral route of the AV extract was less effective than the local and rectal ones.

### 3.14. Aloe vera in Metabolic, Neurological, and Endocrine Diseases

AV has been recognized as a traditional therapy for diabetes, metabolic syndrome, and hyperlipidemia management [220,221]. Preclinical and clinical studies, summarized by Deora et al. (2022) [222], reported that the oral administration of AV is likely effective in improving blood glucose homeostasis and lipid metabolism, which is useful for alleviating diabetic dyslipidemia. The oral administration of AV juice has shown promising results in reducing fasting glycemia and triglycerides in type 2 diabetes mellitus (T2DM) patients, either alone or combined with a conventional antidiabetic drug [223]. Tanaka et al. (2006) [224] attributed the anti-glycemic activity in T2DM to five phytosterols, namely lophenol, 24-methyl-lophenol, 24-ethyl-lophenol, cycloartanol, and 24-methylene-cycloartanol. Devaraj et al. (2013) [225] studied the effects of AV, compared to placebo, on fasting blood glucose, lipid profile, and oxidative stress in subjects with prediabetes/metabolic syndrome. In this case, AV represented an interesting adjunctive strategy to revert the impaired fasting glucose and glucose tolerance observed in conditions of prediabetes/metabolic syndrome. An antidepressant effect of AV in prediabetic patients was also reported by Foadoddini et al. (2020) [226]. The authors demonstrated that the intake of at least 500 mg of AV capsules reduced the depressive state in patients after 8 weeks of treatment. Moreover, the concomitant use of fluoxetine, a selective serotonin reuptake inhibitor (SSRI), determined a reduction in the depressant symptoms in a mouse model [227]. A review on AV and streptozotocin-induced diabetes mellitus has been recently reported by Haghani et al. (2022) [228]. Tabatabaei et al. (2017, 2023) [229,230] reported an in vivo study on streptozotocin (STZ)-induced diabetic adult male Wistar rats. The results of behavioral tests showed that diabetes augmented anxiety/depression-like behaviors, reduced exploratory and locomotor activities, decreased memory performance, and increased stress-related behaviors. These changes in diabetic rats were accompanied by increasing oxidative stress and neuronal loss in the hippocampus. The treatment with AV gel for eight weeks alleviated these deficits related to diabetes, and in some aspects, it was even more effective than insulin. The authors concluded that both interrelated hypoglycemic and antioxidative properties of AV gel are likely involved in improving behavioral deficits and protecting the hippocampal neurons in diabetic mice. Firempong et al. (2023) [231] recently reported the use of AV Burm. f. leaves in the Community of Toase, in the Ashanti Region, Ghana, for the treatment of diabetes mellitus. In a recent study, a new dipyrrole was identified, namely 3,6-dioxo-3,3a,6,6a-tetrahydropyrrolo [3,4-*c*]-pyrrole-1,4-dicarboxamide, which is likely responsible for the antidiabetic activity of AV by showing inhibitory activity against the diabetic drug target dipeptidyl peptidase (DPP) IV in vitro [232]. Finally, AV has been suggested as a safe and effective adjunctive treatment for DFU, a prevalent complication of diabetes that can result in severe consequences [233].

### 3.15. Prebiotic and Probiotic Effects of Involvement of Aloe vera with the Gut Microbiota

Recent scientific studies have suggested that the ingestion of both prebiotics and probiotics can help fight chronic diseases, since colon fermentation produces short-chain fatty acids (SCFAs), which include butyrate, propionate, and acetate, able to influence the energy balance and metabolism of the brain, possessing neuroactive properties [234]. Liu et al. (2021) [235] reported that the administration of AV significantly increased the content of SCFAs, derived from the fecal fermentation of AVPs. The prebiotic effect of AV was also demonstrated by Gullón et al. (2015) [236] since it was able to increase the abundance of *Prevotella*, *Catenibacterium*, *Lachnospiraceae*, and *Coprococcus* and reduce the harmful microbiota, including *Escherichia–Shigella* and *Veillonella*. Ahmed et al. (2023) [237] reported a study on the production of a symbiotic yogurt containing *Lacticaseibacillus rhamnosus* and AV gel. The AV gel considerably enhanced the viability of *L. rhamnosus* during the shelf life, which normally represents a practical limit for a probiotic. AV gel improved the yogurt’s antioxidant and antimicrobial activity, proteolytic content, water-holding capacity, and sensory aspects. The addition of 5% AV gel to probiotic yogurt produced a functional food with 68% desirability that retained its beneficial properties for at least two weeks, under refrigerated storage. Interestingly, natural extracts of AV appear to be potential candidates to re-equilibrate the gut microbiota, which could be a useful feature to be exploited in multiple sclerosis [238] and COVID-19 [121,239,240,241].

### 3.16. Aloe vera Activity in Dentistry and Oral Cavity Disorders

The usefulness of AV in dental caries has been recently reported in a systematic review [242]. Specifically, AV mouthwash demonstrated a significant reduction in plaque and gingival scores and dental caries, comparable to chlorhexidine mouthwash, suggesting it as an alternative for maintaining dental health. Most studies are related to the investigation of the antibacterial activity against *Streptococcus mutans*, the major bacterium responsible for dental caries [243]. Fani & Kohanteb (2012) [244] investigated the inhibitory activities of AV gel on a cariogenic (*S. mutans*), some periodontopathic (*Aggregatibacter actinomycetemcomitans*, *Porphyromonas gingivalis*), and an opportunistic periodontopathogen (*Bacteroides fragilis*) isolated from patients with dental caries and periodontal diseases. *S. mutans* was found to be highly sensitive to AV gel, followed by *A. actinomycetemcomitans.* A recent study by Al-Abdullah et al. (2022) [245] evidenced that AV extract, used as a cavity disinfectant, increases the success rate of the selective caries removal technique of deep carious lesions, more than chlorhexidine. Moreover, a formulation of toothpaste gel containing a mixture of AV and red betel (*Piper crocatum*) extract has effectively proven to lower the growth of the *S. mutans* [246]. A recent randomized controlled trial was carried out to compare the antiplaque and antibacterial efficacy of commercially available mouthwashes containing AV and cetylpyridinium chloride in a 4-day plaque regrowth study. Data demonstrated that AV mouthwashes showed activity similar to that of cetylpyridinium chloride [247]. The randomized clinical trial [248] comparing the effects of AV and probiotic mouthwashes to fluoride mouthwash on *S. mutans,* in plaque around brackets of orthodontic patients, also demonstrated that there was no significative difference in efficacy among the considered mouthwashes in reducing the *S. mutans* plaque level. However, a recent study by Naghsh et al. (2023) [249] on gingivitis, associated with dental plaque, assessed that a mouthwash containing AV and green tea had lower antibacterial effects than the chlorhexidine-containing one. A recent study addressed the potential ability of AV to enhance and accelerate bone repair in oral rehabilitation [250]. Bone formation was studied in non-critical defects of rat tibias, after implantation of a collagen sponge (Hemospon^®^) colonized with mesenchymal stem cells from human dental pulp (hDPSCs) and AV. The results suggest that the combination of Hemospon^®^, AV, and hDPSCs is a form of clinical treatment for the repair of non-critical bone defects that reduces the inflammatory cascade’s effects. The use of AV as an intracanal medicament in the field of endodontics has become noteworthy, thanks to its antibacterial effect against *Enterococcus faecalis* [251]. Indeed, Ghasemi et al. (2020) [252] demonstrated that AV, in contrast to calcium hydroxide, eliminated four and six weeks of biofilm production, showing remarkable antibacterial properties against *E. faecalis* biofilm. The study by Babaee et al. (2012) [253], carried out as a double-blind (case-control) clinical trial on 40 patients with minor aphthous lesions, demonstrated that AV gel is likely to effectively reduce aphthous stomatitis, a recurrent spontaneously healing lesion mostly affecting the lips, soft palate, and throat in children and young adults. It also reduced the wound size and decreased the aphthous wound-healing period. Furthermore, the effectiveness of AV in oral candidiasis is reported [254]. Oral *Candida* infection is an opportunistic fungal infection that leads to acute and chronic infections in the mouth and gastrointestinal tract, and is common among people who are immunocompromised, those with poor oral hygiene, diabetics, and those of advanced age. Jeevitha et al. (2018) [255] analyzed the bioactive components from AV ethanol extract, by gas chromatograph–mass spectrometer, and evaluated the antimicrobial effects on *C. albicans.* The ethanol extract, containing 26 bioactive compounds, showed the highest inhibitory growth against yeast, with an inhibition zone of 23 mm. The minimum inhibitory concentration and the minimum fungicidal concentration of the extracts were also determined, with researchers concluding that AV has potent antifungal activity against *C. albicans.* Rezvaninejad et al. (2022) [256] recently reported a study concerning oral candidiasis. Specifically, they described the comparison of the effects of AV gel and nystatin in *C. albicans*, *C. glabrata,* and *C. tropicalis.* AV gel showed antifungal properties in all tested species. The inhibitory concentrations of AV were much higher than those of nystatin. Moreover, a recent study by Pouyafard et al. (2023) [257] recently demonstrated that a hydroalcoholic extract from AV leaves showed concentration-dependent antifungal activity against *C. albicans*. AV extract at a 75% concentration effectively inhibited the growth of *C. albicans*. Oral submucous fibrosis (OSF) is a potentially malignant disorder characterized by fibrosis of the oral mucosa. A recent systematic review by Chen et al. (2023) [258] summarized the different medicinal interventions available for the management of OSF, including steroids, hyaluronidase, pentoxifylline, lycopene, curcumin, spirulina, AV, omega3, oxitard (capsules), allicin, and colchicine, concluding that AV is the most effective in relieving the symptoms of severe burning sensations. Nigam et al. (2023) [259] carried out a randomized controlled trial on 120 Grade II OSF patients, in order to compare the efficacy of AV, curcumin, and conventional intervention. Both curcumin and AV showed promising results in terms of mouth opening and the reduction in fibrotic bands. Alveolar osteitis (dry socket) is a complication of dental extractions, which is associated with severe pain developing 2 to 3 days postoperatively [260]. The efficacy of AV in this disease was reported by Alhalabi et al. (2022) [261]. The authors demonstrated that the topical intra-alveolar application of AV extract powder after extraction may decrease the incidence of alveolar osteitis (dry socket), improve socket healing, and reduce post-extraction pain. Oral mucositis (OM) is an oral cavity disorder commonly associated with chemotherapy and/or radiation. Mansouri et al. (2016) [262] studied the effect of AV on chemotherapy-induced OM in patients with acute lymphocytic leukemia and acute myeloid leukemia, comparing the effect of an AV solution with mouthwashes, typically recommended by hematologic centers, including normal saline, nystatin, and chlorhexidine. It was demonstrated that there was a significant difference in the intensity of stomatitis and pain. Karbasizade et al. (2021) [263] compared the effects of AV mouthwash, atorvastatin, and placebo on chemotherapy-induced OM in a double-blinded randomized clinical trial on 120 patients. It was observed that about 50% of the placebo patients and patients treated with atorvastatin experienced mucositis, while that value decreased to 2.5% in the group treated with AV mouthwash. Alkhouli et al. (2021) [264] studied the effect of a 70% AV solution in 26 children with acute lymphoblastic leukemia for the prevention of chemotherapy-induced OM in comparison to a 5% sodium bicarbonate solution. The application of the AV solution was effective in the prevention of OM and reduction in OM severity. Sahebjamee et al. (2015) [265] reported the results of a triple-blind randomized clinical trial comparing the efficacy of AV and a benzydamine mouthwash in the alleviation of radiation-induced OM, in a study on 26 head and neck cancer patients, demonstrating that AV mouthwash was as efficient as benzydamine at reducing the severity of radiation-induced OM.

## 4. *Aloe vera* in Cosmetics and Sanitizers

AV is employed in personal care products, such as soaps, cleansers, sunscreens, face antiaging treatments, lotions, and tissue paper coatings, in concentrations ranging from 1% to 98% [62,266,267]. It is also present in the formulation of some moisturizers, even though the rationale for its inclusion has not been based on controlled studies or evidence-based meta-analyses of clinical trials [268,269]. AV gel is also present in non-alcoholic hand sanitizers, since it contains 99% water and is an excellent natural humectant and emollient [42,270]. AV gel in hand sanitizers can also be used to counteract the burning sensation of essential oils through its wound-healing ability [271].

## 5. *Aloe vera* as a Preservative for Foods

The food industry faces the need to preserve the safety and quality of fresh fruits, vegetables, and fresh-cut products. Edible coatings represent an efficient strategy to maintain the freshness of these products, by preventing postharvest losses and extending their shelf life. They can also be used in conventional packaging as an alternative to modified atmosphere packaging. AV gel is a natural hydrocolloid and has been applied, in recent years, on fruits (grapes and plums) and vegetables as an edible coating [272,273]. It may behave as a semipermeable barrier for water vapor and gases, reducing the ripening process of the fruit, and thus preserving weight, firmness, and valuable compounds. Its use as an edible coating on fruits and vegetables, with or without additives, has been recently and widely reviewed [274]. Due to its antioxidant and antimicrobial properties, it may also represent an interesting material for enhancing the shelf life of fruits and vegetables [275]. Hassanpour (2015) [276] reported that raspberry fruits coated with AV gel showed higher levels of antioxidant capacity, total phenol, total anthocyanin, and antioxidant enzymes during storage periods. A recent study was carried out by Partoazar et al. (2023) [277] on strawberries by comparing the cytotoxicity and antibacterial effects of zeolite/zinc oxide nanocomposite (Zeo/ZnONC) alone and with AV gel (Zeo/ZnONC-AG) on *Shigella sonnei* and *Shigella flexneri* and their effects on the durability of strawberries. The presence of AV gel determined a reduction in MIC and MBC values against both bacteria. The growth of mold on the surface of strawberries treated with Zeo/ZnONC-AG showed a delay with the increase in ZnO concentration, at refrigerator temperature. In addition, the use of AV gel with lemongrass essential oil, as an edible coating, has been shown to considerably enhance the productivity of strawberry fruits and has been suggested for commercial scale [278]. Recently, AV gel treatment in combination with CaCl_2_ has been shown to effectively mitigate internal browning and senescence scald in “conference” pears [279]. The antimicrobial packaging with zinc oxide nanocomposites containing AV gel may represent a beneficial solution for preserving and improving the quality, safety, and shelf life of fresh meat products, as demonstrated in the packaging of chicken fillets, due to their antimicrobial activity against *Salmonella typhi* and *Salmonella paratyphi* A [280]. Kouser et al. (2023) reported the development of a bioactive edible film using carrageenan and AV gel, which demonstrated antibacterial activity against *E. coli* and reduced the lipid oxidation in, and enhanced the microbial quality of, Kalari cheese, a popular Himalayan cheese [281]. Finally, the use of AV with chitosan improved the antioxidant, antimicrobial, thermal, and barrier properties of chitosan in food [282].

## 6. Composition of *Aloe vera* and Studies on the Single Components (Commercially Available)

AV can be considered as a large container of natural substances with high biological and nutritional value, with more than a hundred functional substances isolated by this plant [283], which have been extensively summarized by Choi et al. (2003) [284] and Babu & Noor (2020) [285]. They can be divided into three major groups: anthraquinones, phenolic substances, and glycosides found in the leaf cuticle, which represent the main components of the so-called “anthraquinone latex”; high-molecular-weight polysaccharides, mainly represented by acemannans, galactomannans, and glucomannans, which are abundant in the parenchymatous tissue located within the leaf; and numerous biomolecules of considerable nutritional and functional importance, such as monosaccharides, disaccharides, amino acids, glycoproteins, organic acids, phytosterols, phospholipids, enzymes, saponins, lignins, enzymes, vitamins, and minerals [286,287,288,289]. The most representative are shown in Table 5. The component undoubtedly present in the largest quantity is water, which constitutes, on average, about 96–97% of the fresh weight. Water-soluble vitamins in AV include those of the B group (B1, B2, B3, B6, B12) and vitamin C; fat-soluble vitamins include A, D, and E. AV also contains many minerals essential for the human body, such as iron, chromium, phosphorus, magnesium, manganese, potassium, copper, sodium, and, furthermore, calcium and zinc, which are two essential elements for building tissues and healing wounds [290]. AV contains amino acids essential for humans, including phenylalanine, isoleucine, leucine, lysine, methionine, threonine, and valine, and non-essential amino acids, such as aspartic acid, glutamic acid, alanine, L-arginine (particularly abundant), glycine, glutamine, hydroxyproline, histidine, proline, and serine, as well as cysteine and thyroxine, which are considered semi-essential [291]. Finally, enzymes are represented by cellulase, carboxypeptidase, catalase, amylase, and oxidase, but also lipase and protease, which are involved in the digestion of fats and proteins, and bradykinase, which can interfere with tissue inflammation processes [292,293]. Particularly abundant are triterpenoids (lupeol) [294] and sterols, such as cholesterol, campsterol, and β-sitosterol [295]. Moreover, flavonoids (resveratrol, quercetin, genistein, and naringin), phenols (resveratrol, thymol, D-catechin, and pyrocatechol), phenolic acids (chlorogenic acid, sinapic acid, caffeic acid, coumaric acid, vanillic acid, ferulic acid, syringic acid, and gallic acid), carboxylic acids (cinnamic acid) [83], lectin, chromones (umbelliferone), coumarins (esculetin), cellulase, catalase, and superoxide dismutase are present, as well [296]. The variation in the concentration of these chemical constituents depends on different factors, such as the part of the plant used, the stage of growth, the extraction process, the solvent, and the plant source. Although generally beneficial, some of these phytochemicals may also be responsible for some toxic effects [297].

### 6.1. Anthraquinones and Anthrones

Anthraquinones (9,10-dioxoanthracenes or anthracenediones) represent an important class of natural and synthetic compounds with a wide range of applications. Anthrones (9,10-dihydro-9-oxo-anthracene) are also anthracene derivatives, but they bear only a carbonyl group at the C-9 position and lack the carbonyl group at the C-10 position. Both of them are, sometimes, indiscriminately called anthraquinones and/or anthrones, given their similar structural formula. However, only aloin is an anthrone. Aloin is also a glycoside but is generally included in the class of anthraquinones and anthrones. In addition to the use of anthraquinones as colorants, these compounds have been used, for centuries, for medical applications as laxatives, antimicrobial, and anti-inflammatory agents [298]. Current therapeutic indications also include constipation, arthritis, multiple sclerosis, viral infections, and cancer [299,300,301]. They have also demonstrated usefulness as photosensitizers in photodynamic cancer therapy [302,303].

#### 6.1.1. Aloe-Emodin

Emerging evidence suggests that aloe-emodin (C_15_H_10_O_5_, 1,8-dihydroxy-3-hydroxymethyl-anthraquinone, 3-hydroxymethyl-chrysazin, 1,8-dihydroxy-3-hydroxymethyl-9,10-anthracenedione) exhibits many pharmacological effects, including anticancer, antiviral, anti-inflammatory, antibacterial, antiparasitic, neuroprotective, and hepatoprotective activities. It is suggested for the treatment of various diseases, such as viral influenza, inflammation, sepsis, Alzheimer’s disease, Parkinson’s disease, Huntington’s disease, glaucoma, malaria, liver fibrosis, psoriasis, T2DM, growth disorders, and several types of cancers [304,305]. Moreover, lipid-lowering effects, which play a role in cardiovascular diseases and vascular calcification, have been demonstrated for aloe-emodin [306,307,308,309]. Bai et al. (2018) [310] reported an in vivo study in adult male Wistar rats, demonstrating that aloe-emodin significantly shortened the QT interval, action potential duration at 90% repolarization, and resting membrane potential, which were markedly elongated by a high-fat diet. Furthermore, it significantly inhibited pro-arrhythmic miR-1 in the hearts of high-fat-diet rats. In vitro, the decrease in miR-1 expression levels resulted in an increase in Kir2.1 protein levels. The anticancer activity of aloe-emodin was demonstrated in diverse cell lines, specifically U87 malignant glioma cells, SJ-N-KP neuroblastoma cells, SVG (transformed glial) cells, U-373MG cells (human glioma), MDA-MB-453 and MCF-7 breast cancer cells, MGC-803, SGC-7901, BGC-823, and MKN45 gastric cancer cells, KB oral mucosa cells, HeLa cervical cancer cells, and TE1 esophageal cancer cells [311,312]. In the study by Tabolacci et al. (2015) [313], the antineoplastic activity of aloe-emodin in metastatic human melanoma SK-MEL-28 and A375 cell lines and melanospheres (primary stem-like cells) was investigated. The treatment with aloe-emodin determined a notable increase in the trans-amidating activity of trans-glutaminase. Aloe-emodin also displayed an immunomodulatory effect and significantly reduced melanophores’ proliferation, invasive potential, and stemness. Recently, a peptide-mediated targeted delivery of aloe-emodin has been studied against the human breast adenocarcinoma cell line SKBR3 [314], which overexpresses the human epidermal growth factor receptor 2 (HER2) receptor [315], and its bioconjugate efficacy was compared to the compound itself towards a non-small cell lung cancer cell line, A549. Results demonstrated the higher activity of the new conjugate with respect to aloe-emodin alone (prepared by oxidation with a ferric chloride of commercial aloin from Curacao aloe—52% purity, lot SLBC4749V—purchased from Sigma-Aldrich, St. Louis, MO, USA), thus suggesting a promising specific activity towards HER2-expressing cells, coupled with an enhanced water solubility and higher cytotoxicity [314]. Aloe-emodin mechanisms for anticancer activity consist of the inhibition of cell growth and proliferation, cell cycle arrest, initiation of apoptosis, and antimetastasic and antiangiogenic effects [311,316]. Specifically, studies evidenced, among other things, the suppression of cancer, by targeting the mTOR complex 2, as demonstrated in prostate cancer cells [317]; through the p53-dependent and p21-dependent apoptotic pathway in human hepatoma cell lines [318]; by the inhibition of AKT and ERK phosphorylation in TE1 esophageal cancer cells [319]; and by the inhibition of the ERK/MSK1 and AKT/GSK3β signaling pathways in epidermal growth factor (EGF)-induced neoplastic cell transformation by inhibiting the ERK/MSK1 and AKT/GSK3β signaling pathways [320]. A possible mechanism of the anticancer mechanism of aloe-emodin has been recently underlined by Meng et al. (2022) [321] through the inhibition of CYP1B1 enzyme. Su et al. (2023) [322] studied the anti-inflammatory properties of aloe-emodin, demonstrating its interesting activity in ameliorating cecal ligation and puncture-induced sepsis in vivo, in specific pathogen-free male and female C57BL/6 mice. Furkan et al. (2017) [323] demonstrated that aloe-emodin acts as an inhibitor of hemoglobin aggregation in vitro; thus, it is potentially useful for the treatment of neurodegenerative diseases like Alzheimer’s, Parkinson’s, and Huntington’s. The antiviral activity of aloe-emodin has been related to its interaction with galectin-3, a beta-galactoside-binding lectin, involved in viral infections, including SARS-CoV-2 [324]. The treatment with aloe-emodin was reported to upregulate Gal-3 expression in infected cells, leading to an increased expression of antiviral genes such as IFN-β, IFN-γ, protein kinase R (PKR), and 2′,5′-OAS via the JAK/STAT signaling pathway. Galectin-3 inhibited influenza A virus replication, as well [325]. Zeng et al. (2019) [326] studied the inhibitory effect of aloe-emodin on tyrosinase activity by spectroscopic and molecular docking techniques. It was suggested that aloe-emodin spontaneously binds tyrosinase at one binding site in the hydrophobic cavity. Ma et al. (2020) [327] suggested the use of aloe-emodin as a natural effective photosensitizer in antimicrobial photodynamic therapy against oral infections of drug-resistant *C. albicans*. Pharmacokinetic studies have demonstrated that aloe-emodin has a poor intestinal absorption, short elimination half-life, and low bioavailability. Researchers have tried to overcome the latter by structural modifications [328] and the use of nanocarrier systems [329]. The strong limit of aloe-emodin is represented by its adverse effects, recently reported by an increasing number of published studies. In the early 2000s, exposure of human skin fibroblasts to aloe-emodin and ultraviolet radiation was demonstrated to cause significant phototoxicity [330]. In recent years, the primary toxicities reported are hepatotoxicity and nephrotoxicity, which are of wide concern worldwide [331]. Quan et al. (2019) [332] reported that aloe-emodin (purchased from Chroma-Biotechnology Co., Ltd. (Chengdu, China)) induced hepatotoxicity by activating the NF-κB inflammatory pathway and P53 apoptosis pathway in vivo, using zebrafish Tg (fabp10: EGFP) as an animal model. However, Galli et al. (2021) [333] demonstrated that aloe-emodin is not genotoxic in an in vivo comet test.

#### 6.1.2. Emodin

Emodin (1,3,8-trihydroxy-6-methylanthraquinone, 3-hydroxy-6-methyl-chrysazin, C_15_H_10_O_5_) is the position isomer of aloe-emodin. It has demonstrated several pharmacological properties, such as immunosuppressive, antibacterial, antifungal, antiviral, hepatoprotective, antioxidative, and antitumor [334]. Emodin is also studied for its activity on cardiovascular disease and atherosclerosis prevention [335,336]. Existing reports indicate that emodin is characterized by proapoptotic, pro-oxidative, and antiangiogenic effects [337]. Liu et al. (2011) [338] demonstrated that emodin significantly downregulated NF-κB DNA-binding activity, survivin, and MMP-9. Moreover, the expression of cleaved caspase-3 was upregulated in pancreatic SW1990 cancer cell lines after treatment with emodin. Trybus et al. (2017) [337] demonstrated that emodin promotes the death of cervical cancer cells through changes in the lysosomal compartment. As demonstrated by Meng et al. (2022) [321], emodin also inhibits CYP1B1, even though the inhibitory effect is lower than that of its position isomer aloe-emodin. However, the antiproliferative and anti-carcinogenic properties of emodin are likely accompanied by potential toxicity, which may be increased by the drug delivery systems in terms of bioavailability [339].

#### 6.1.3. Aloin

Aloin (C_21_H_22_O_9_) is the C-glucoside of aloe-emodin and an anthrone. It is a yellow aromatic organic compound, consisting of two diastereoisomers, aloin A (barbaloin) and aloin B (isobarbaloin), which is localized in the outer rind of the aloe plant and constitutes up to 30% of the aloe plant’s dried leaf exudates. Aloins A and B should be considered two different compounds; nevertheless, even today, several authors continue to describe the activities of aloin as a singular compound [340]. Aloin A is the (10*S*)-10-glucopyranosyl-1,8-dihydroxy-3-(hydroxymethyl)-9(10*H*)-anthracenone or 10-C-β-D-glucopyranosyl-1,8-dihydroxy-3–(hydroxymethyl)-9-anthracenone, whereas aloin B is the (10*R*)-10-glucopyranosyl-1,8-dihydroxy-3-(hydroxymethyl)-9(10*H*)-anthracenone. However, Cardarelli et al. (2017) [341] reported the contrary, stating that aloin A is the (10*R*) enantiomer and aloin B is the (10*S*) one. Studies of aloin’s biosynthesis indicate that aloin B (the C_10_,C_1′_:R,S diastereomer) is preferentially formed. The nonenzymatic conversion to aloin A (the C_10_,C_1′_:S,S diastereomer) is thought to result in the mixture of aloin A and aloin B observed for naturally derived aloin [342]. In recent decades, several studies were reported on aloin, demonstrating its anti-inflammatory [343], antioxidant [344], antitumor, and anti-organ-injurious activities [345,346], as well as neuroprotection [347] and osteoclastogenesis [348,349,350]. Boudreau et al. (2017) [351] suggested that aloin can induce pathological changes and modulate the composition of the microbiota in the large intestine of F344/N male rats. In inflammatory-associated diseases, aloin is able to attenuate LPS-induced inflammation by inhibiting ROS-mediated activation of the JAK1-STAT1/3 signaling pathway, thereby inhibiting the nuclear translocation of STAT1/3 in RAW264.7 cells [352]. Several studies concerning the anticancer activities of aloin have been reported, and they are summarized in Table 6. Hu et al. (2022) [353] described an in vitro study on CAL-27 oral squamous cell carcinoma (OSCC) and evaluated the classical AKT/mTOR signal transduction pathway. Aloin promoted apoptosis and autophagy and downregulated the protein expression of p62, whereas Beclin-1 and LC3-I/LC3-II protein expressions were upregulated. Further investigations suggested that aloin could block the activation of the AKT/mTOR pathway. Tao et al. (2019) [354] reported that aloin induced HGC-27 human gastric cancer cell apoptosis by downregulating expressions of High Mobility Group Box 1 (HMGB1) and RAGE, inhibiting HMGB1 release and suppressing rhHMGB1-induced activation of the AKT/mTOR-P70S6K and ERK-P90RSK-CREB signaling pathways. Wang et al. (2020) [355] demonstrated that aloin inhibited the proliferation and migration of HGC-27 and BGC-823 gastric cancer cells by regulating NOX2-ROS-mediated pro-survival signal pathways. However, the mechanisms affected by aloin in gastric cancer are complex and multifactorial; thus, they cannot be explained by only unilateral factors and/or single targets. Gao et al. (2022) [356] suggested that aloin induces cell apoptosis and regulates the PI3K/AKT signaling pathway in gastric cancer. Chen et al. (2023) [357] indicated that aloin promotes MGC-803 gastric cancer cell apoptosis through the miR-5683/HMGB1 axis. Recently, Ahmed et al. (2023) [358] studied the antitumor activity of aloin on estrogen receptor-positive (T47D) and triple negative (MDA-MB-231) breast cancer cell lines, in comparison with the standard anthraquinone doxorubicin (Dox). Aloin inhibited both types of cancer cells’ growth, in a time- and dose-dependent manner, with a more pronounced effect in the 72 h exposure regimen, and mostly in the ERα+ breast cell line. The data suggested that autophagy can be one of the mechanisms underlying aloin cytotoxicity in breast cancer cells that evade apoptosis through genetic mutations in p53. Molecular docking and ADMET studies by Mani et al. (2023) [359] indicate that aloin may behave as a potential anti-breast cancer agent by targeting the ER. Li et al. (2020) [360] reported that in human melanoma A375 cells, aloin promotes cell apoptosis by downregulating HMGB1 expression at the transcriptional level, preventing its translocation to the cytoplasm and interaction with TLR4, which indeed blocks HMGB1-mediated ERK activation. Sun et al. (2020) [361] demonstrated that the association of aloin with metformin increased the antiproliferative effect in hepatocellular carcinoma cells, via PI3K/AKT/mTOR-mediated apoptosis and autophagy. This combination enhanced the expression of Beclin-1, LC3-II, and ATG8 in HepG2 and Bel-7402 cells, in comparison with the untreated group, and downregulated P62. Jassi et al. (2023) [362] reported that the combination of CPT-11, a drug used in colorectal cancer treatment, with aloin enhanced the antitumor activity of CPT-11. This combination reduced cell viability and induced apoptosis, both in vitro and in vivo. Moreover, miRNA-133b was upregulated, whereas IGF1R and its downstream MEK/ERK and PI3K/AKT/mTOR pathways were downregulated. Moreover, aloin acts as an inhibitor of tyrosinase, the enzyme that converts L-tyrosine into L-dihydroxyphenylalanine (L-DOPA), which represents one of the key enzymes in synthesizing melanin polymers [363]. Furthermore, aloin has been shown to selectively inhibit the proteolytic and deubiquitinating activity of papain-like protease (PL^pro^) of SARS-CoV-2 in vitro [364]. Recent studies are mostly focused on the activities of aloin A rather than aloin B. Mitra et al. (2022) [365] have recently reported a review summarizing the activities attributed to aloin A, such as antioxidant, anti-inflammatory, antidiabetic, anticancer, antimicrobial, antiviral, and immunity-boosting actions. Moreover, the activities of aloin A in attenuating pulmonary fibrosis through the TGF-β1/Smads/p38 pathway [366] and promoting the osteogenic differentiation of human bone marrow mesenchymal stem cells (hBMSCs) by the regulation of the Wnt/β-catenin signaling pathway [367] have been recently reported, as well as its neuroprotective effect in rats [368]. Cao et al. (2017) [369] reported an antiarrhythmic activity of aloin A by measuring the cardiac action potentials and ionic currents in isolated rabbit ventricular myocytes using a whole-cell patch-clamp technique and studying its antiarrhythmic effect in Langendorff-perfused rabbit hearts. The effect of aloin A in gestational diabetes mellitus (GDM) was studied by Wang et al. (2020) [370] in vivo in db/+ diabetic mice treated with metformin. Aloin A significantly lowered blood glucose levels and enhanced insulin levels in GDM mice. Furthermore, it reduced the inflammatory response and ROS levels in liver. The hypoglycemic and hypolipidemic effects on GDM mice were suggested to have been exerted, at least in part, via the modulation of the AMP-activated protein kinase (AMPK)/peroxisome proliferator-activated receptor gamma coactivator 1-alpha (PGC-1α) signaling pathway. Alhadrami et al. (2023) [371] reported that aloin A is able to inhibit SARS-CoV-2 replication by targeting its binding with ACE2. In vitro assays were carried out against SARS-CoV-2 proteases (i.e., main proteases M^Pro^ and PL^Pro^) showing weak to moderate activity (IC_50_ = 68.56 ± 1.13 µM and 24.77 ± 1.57 µM, respectively). Aloin A was also able to inhibit the replication of SARS-CoV-2 in Vero E6 cells efficiently, with an IC_50_ value of 0.095 ± 0.022 µM. Recent studies have addressed the stability of aloin A on the basis of the pH of different solutions, which is important for the evaluation of the mechanism of action of this compound in diverse parts of an organism, as well as the prediction of pharmacologically relevant parameters, such as absorption, distribution, metabolism, and excretion [372]. Aloin is also effective in combined allergic rhinitis and asthma syndrome (CARAS), and its mechanism has been recently evaluated in ovalbumin-induced CARAS mice, resulting in the modulation of the MAPK signaling pathway [373].

#### 6.1.4. Rhein

Rhein (4,5-dihydroxy-9,10-dioxoanthracene-2-carboxylic acid, cassic acid) is a pharmacologically active component primarily found in *Rheum palmatum* L. (Chinese rhubarb), used traditionally as a purgative and cathartic. It has also shown antiseptic, liver stimulant, diuretic, stomachic, anticholesterolemic, anticancer, neuroprotective [374], antioxidant [375], anti-obesogenic [376,377], antiamebic [378], and wound healing [379] activity. Rhubarb is one of the most ancient and important herbs in traditional Chinese medicine (TCM) and a popular food that represents one of the most effective laxatives, widely used in the treatment of intestinal constipation in Europe and throughout the world [380]. Xu et al. (2017) demonstrated that rhein had a potential neuroprotective role in traumatic brain injury (TBI). The combination of rhubarb and emodin has been suggested for the treatment of UC [381]. Cheng et al. (2020) [382] identified rhein as one of the metabolites of sennoside A that accumulated most over time. Cell culture experiments suggested that apoptosis and autophagy induced by rhein are the likely mechanisms of the chronic toxicity of rhubarb anthraquinones.

**Table 6:** Preclinical studies of aloin in cancer.

Cancer Type	Cell Lines	Aloin Activity	Ref.
**Oral Squamous Cell Carcinoma (OSCC)**	CAL-27	Promotes apoptosis and autophagy downregulation of p62 protein expression; upregulates Beclin-1 and LC3-I/LC3-II protein expression; blocks AKT/mTOR pathway activation	[353]
**Gastric Cancer**	HGC-27	Induces apoptosis by downregulating expressions of High Mobility Group Box 1 (HMGB1) and RAGE, inhibiting HMGB1 release, and suppressing rhHMGB1-induced activation of AKT/mTOR-P70S6K and ERK-P90RSK-CREB signaling pathways	[354]
**Gastric Cancer**	HGC-27 and BGC-823	Inhibits the proliferation and migration of gastric cancer cells by regulating NOX2-ROS-mediated pro-survival signal pathways	[355]
**Gastric Cancer**	NCI-N87	Induces cell apoptosis and regulates the PI3K/AKT signaling pathway	[356]
**Gastric Cancer**	MGC-803	Promotes apoptosis through the miR-5683/HMGB1 axis	[357]
**Breast Cancer**	T47D (estrogen receptor-positive) and MDA-MB-231 (triple negative)	Autophagy suggested as one of the mechanistic modes of aloin cytotoxicity through genetic mutations in p53	[358]
**Melanoma**	A375	Promotes cell apoptosis by downregulating HMGB1 expression at the transcriptional level, preventing its translocation to the cytoplasm and interaction with TLR4, which indeed blocks HMGB1-mediated ERK activation	[360]
**Hepatocellular Carcinoma**	HepG2 and Bel-7402	In association with metformin: —increased antiproliferative effect via PI3K/AKT/mTOR-mediated apoptosis and autophagy —enhanced the expression of Beclin-1, LC3-II, and ATG8 and downregulated P62	[361]
**Colorectal Cancer**	LoVo, SW620, and Caco2 (in vitro); six-week-old male NU/NU nude mice (in vivo)	In combination with CPT-11: —enhanced the antitumor activity of CPT-11 —reduced cell viability and induced apoptosis, both in vitro and in vivo —upregulated miRNA-133b and downregulated the IGF1R and its downstream MEK/ERK and PI3K/AKT/mTOR pathways	[362]

#### 6.1.5. Chrysophanol

Chrysophanol, also known as chrysophanic acid (1,8-dihydroxy-3-methylanthracenedione), is an anthraquinone present in AV and is one of the most important anthraquinone components isolated from plants of the *Rheum* genus [131,383]. It exerts a number of beneficial effects, such as anti-inflammation [384], anticancer [385,386], antidepressive, and antioxidant [387] effects, and neuroprotectant activity [388]. It has been shown to alleviate acute lung injury caused by *Klebsiella pneumoniae* infection by inhibiting pro-inflammatory cytokine production [389]. Several recent studies have addressed the exploration of its activities and possible toxicity [390,391]. A protective activity against neural fibrosis [392], as well as the facilitation of long-term neurological recovery, contributing to repair and regeneration after ischemic stroke [393], have been reported.

### 6.2. Glycosides

#### 6.2.1. Glycosylated Chromones

Glycosylated chromones are mostly present in the plant leaf. Among these, aloesin, aloeresin A, and isoaloeresin D are the most important that have been isolated and identified from AV [44]. Aloeresin E, aloeresin D, rabaichromone, and aloeresin K have been reported as antioxidants, even though it is the concentrations of chromones that determine whether they act as pro-oxidants or antioxidants [289,343,394]. Handayani et al. (2023) [395] reported the anti-collagenase and anti-elastase activities of aloesin, isoaloeresin D, and 7 methyl ether 2′ feruloylaloesin by in silico studies of their interaction with collagenase enzyme (2Y6I). A few studies also report the importance of a less-studied glycoprotein isolated from AV gel, specifically a C-glucosyl chromone, called alprogen, which has antiallergic, anti-inflammatory properties, and is also useful in periodontitis [396,397,398]. Alprogen can help fix damaged insulin cells in the pancreas, improve the working of insulin, and reduce high blood sugar levels; thus, it is useful for the treatment of T2DM [399,400]. It has also been described for its anticancer activity [301,401]. Other minor chromone C-glycosides were described by Lv et al. (2008) [289].

##### Aloesin

Aloesin (2-acetonyl-8-β-D-glucopyranosyl-7-hydroxy-5-methylchromone) is a C-glucosyl-7-hydroxychromone that inhibits the hyperpigmentation induced by UV radiation [402]. It is a bioactive constituent of *Aloe* spp. used primarily in cosmetic products. It is a chromone derivative that was shown to modulate melanogenesis via the competitive inhibition of tyrosinase; thus, it was suggested as a pigmentation-altering agent for cosmetic or therapeutic applications [403]. A high aloesin concentration is found in AV’s photosynthetically active outer cortex (the thick epidermis with cuticle that corresponds to ~31% of the leaf weight) that is often discarded as by-product, with no commercial value [98]. Whaedi et al. (2017) [404] demonstrated that aloesin, whose source (commercial or extracted from AV) was not disclosed, promotes wound healing in vivo, in a mouse model, and skin cell regeneration and migration in vitro. Its involvement in cell migration, tissue development, angiogenesis, and cytokine release has been suggested, and these effects are likely due to the activation of the MAPK/Rho and Smad signaling pathways. Aloesin inhibits the enzymatic activity of beta-secretase (BACE1), suggesting its involvement in the neurodegenerative process that leads to Alzheimer’s disease [289]. The use of aloesin, alone or in a standardized blend with *Aloe* polysaccharides (a composition called Loesyn), significantly reduced the glycosylated hemoglobin, the fasting blood glucose, and fructosamine and plasma insulin levels in humans. Thus, it has been suggested as a potential nutraceutical to manage the systemic oxidative stress and/or high blood glucose of diabetes [405]. The study by Zhang et al. (2023) [406] showed that aloesin (95% in purity, obtained from the Xi’an Yunyue Biotechnology Co., Ltd., Xi’an City, Shanxi Province, China) exerts interesting antifungal properties against *Magnaporthe oryzae*, the major pathogen of rice crops, significantly inhibiting *M. oryzae* spore germination and appressorium formation. Studies on enzyme activity showed that aloesin inhibited the activities of polyketolase (PKS), laccase (LAC), and chain-shortening catalytic enzyme (Aayg1), which represent key enzymes in melanin synthesis. Aloesin is a strong inhibitor of tyrosinase activity as well, in a dose-dependent way [405,407]; thus, it could be useful in the management of hyperpigmentation [408]. Particularly, aloesin inhibits L-DOPA oxidation and showed a better affinity than kojic acid, arbutin, etc., but it poorly penetrates the stratum corneum due to its hydrophilicity and high molecular weight, and needs novel delivery systems to be more effective. It also upregulates cyclin E-dependent kinase activity [409]. Yimam et al. (2013, 2014) [410,411,412] studied an AV-based formulation (called UP780), a standardized composition of aloesin formulated with an AV inner leaf fillet (Qmatrix), which significantly reduced HbA1C, fasting blood glucose, fructosamine, and plasma insulin levels in humans, and improved impaired glucose levels and insulin resistance in high-fat diet-induced and db/db non-insulin-dependent diabetic mouse models. UP780 can also increase the production of adiponectin, an adipocyte-derived plasma protein exclusively produced by fat cells, whose blood levels inversely correlates with insulin sensitivity [413]. Furthermore, molecular modelling studies suggested the potential of some chromones from AV, such as SARS-CoV-2 M^pro^ and spike protein inhibitors [414]. Aloesin also exhibits antioxidant activity and inhibits important enzymes in the regenerative process, including COX-2 and thromboxane A2 synthetase [415]. Amongst the studied aloesin derivatives, isorabaichromone, feruloylaloesin, and *p*-coumaroylaloesin showed potent antioxidative activity, as demonstrated by DPPH assay [416].

##### Aloeresin A

Aloeresin A [(2*S*,3*R*,4*S*,5*S*,6*R*)-4,5-dihydroxy-6-(hydroxymethyl)-2-[7-hydroxy-5-methyl-4-oxo-2-(2-oxopropyl)chromen-8-yl]oxan-3-yl]-(*E*)-3-(4-hydroxyphenyl)prop-2-enoate belongs to the glycosylated chromones and was recently under investigation for its potential antioxidant activities. Breaud et al. (2022) [417] indicated that the antioxidant activity of AV could be ascribed to this compound, together with coumaroylaloesin, based on LC-MS phytochemical profiling. Recent in silico studies by Roshni et al. (2023) [418] have suggested its use for effectively treating keratitis, which may provoke corneal damage and vision loss, as a component of lens care solutions to prevent contact lens-mediated microbial keratitis caused by *P. aeruginosa* and *S. pneumoniae*.

#### 6.2.2. Glycosylated Pyran-2-Ones

Aloenin and aloenin B are glycosylated pyran-2-ones obtained from AV. There are few studies regarding these compounds. However, recent findings suggest the potential involvement of this compound in several biological activities. Aloenin (or aloenin A) has been suggested as a potential inhibitor of pancreatic lipase in vitro, acting in a competitive manner. This finding was supported by molecular docking studies, thus suggesting that the anti-hyperlipidemic effects of AV on pancreatic lipase can be attributed in part to the presence of this compound [419]. Its presence has also been suggested to be related to the anticonstipation effects [204]. Aloenin B has been suggested as a potential inhibitor of P-glycoprotein (P-gp), which is one of the drug transporters that determine the uptake and efflux of a range of drugs and is involved in cancer and multidrug resistance (MDR) [420].

### 6.3. Coumarins and Isocoumarins

Feralolide is a dihydroisocoumarin isolated from the methanolic extract of AV resin and other species belonging to the Aloe genus. A recent study demonstrated antioxidant effects together with a potent urease and weak α-glucosidase enzyme inhibition [421]. Additionally, the compound exerted concentration-dependent antiproliferative effects on breast cancer cells (MDA-MB-231) and ovarian cancer cells (SKOV-3) [422]. Recently, Khan et al. (2023) [423] demonstrated the cholinesterase enzymes’ inhibitory activity of feralolide and its antiamnesic effects in healthy albino mice, thus suggesting its use for treating memory dysfunction in Alzheimer’s disease. Esculetin is a coumarin derivative found in AV. It has been recognized as a promising chemotherapeutic agent, as it inhibits proliferation and induces apoptosis in human cancer cells. In addition, it has been described for its anti-inflammatory, antioxidant, antibacterial, antidiabetic, and antiviral potential, even against SARS-CoV-2 [424].

### 6.4. Aloe vera Polysaccharides (APs)

AV gel consists mainly of water, but a high percentage of polysaccharides (aloe polysaccharides, APs) are also present [425], mainly as acetyl polysaccharides. They are indigestible bioactive polysaccharides but can be fermented by colonic microbiota and constitute most of the dry matter of AV. The polysaccharide composition is not always the same and is principally related to leaf morphology, being, generally, mannose connected by β-1,4-glycosidic bonds [426]. The molecular weights of these polysaccharides vary between 30 and 50 kDa; however, in some circumstances, they can reach 1000 kDa [427]. Several studies on APs have demonstrated their antioxidant, immune-regulation, anti-obesity, and anticancer activities [428]. Kang et al. (2014) [429] reported a study on purified APs that strongly scavenged radicals, including DPPH and hydroxyl and alkyl radicals in vitro. APs also showed a protective effect against 2,2′-azobis (2-amidinopropane)dihydrochloride (AAPH)-induced oxidative stress and cell death in Vero cultures. Moreover, an in vivo study in a zebrafish model demonstrated the APs’ antioxidant activity, as well. Several studies indicate that APs are implicated in gut microbiota modulation, with a prebiotic activity [69,430], and it is generally believed that the polysaccharides present in the AV gel are responsible for the immunomodulatory effects, since they can alter several interactions among the cells of the immune system. Liu et al. (2021) [431] reported that APs may ameliorate acute colitis in mice via the Nrf2/HO-1 signaling pathway by protecting the intestinal barrier in acute UC mice. They were able to scavenge free radicals in vitro and in vivo and exert antioxidant and anti-inflammatory effects, both in serum and in the colon. Moreover, treatment with APs effectively increased short-chain fatty acids (SCFAs) production, and the I expressions of ZO-1, occludin, Nrf2, HO-I, and NQO1 were improved. Cui et al. (2014) [432] attribute a potent protective effect against chronic alcohol-induced liver injury to APs, as demonstrated in a chronic alcohol-feeding mouse model, thus suggesting them as potent food supplements in the prevention of alcoholic liver disease (ALD). The hepatoprotective effect is likely associated with its antioxidant capacity and ability to accelerate lipolysis and inhibit the inflammatory response. A recent study found that *Aronia melanocarpa* Elliot anthocyanins, also known as raspberry, in combination with APs, produced a better remission effect on ALD in mice by activating the PI3K/AKT signaling pathway and promoting the expression of Nrf2 and HO-1, compared with the single-component administration [433]. Among polysaccharides, acemannan is the most important and the most studied, and several properties have been ascribed to this AP [434], as detailed below. The immunomodulatory activity is generally ascribed to acemannan [435]; however, a paper by Pugh et al. (2001) [436] suggests the existence of another polysaccharide, called aloeride, which could be responsible for this activity.

#### Acemannan

Acemannan represents a “storage” polysaccharide, which is not a single and unique compound (from which the name “acemannans” is derived) [437]. It consists of glucose and mannose units linked by 1,4-beta glycosidic bonds and is mainly composed of large amounts of partially acetylated mannose units (Man > 60%), followed by glucose (Glc~20%) and, to a minor extent, galactose (Gal < 10%). Structurally, the acemannan polysaccharide is represented by a single chain of β-(1→4) mannose with β-(1→4) glucose inserted into the backbone; α-(1→6) galactose units may also be found as side chains [438,439]. These discrepancies in glucose–mannose ratios are visible in the different species, depending on the seasonality of the plant from which they are obtained, and the different methods of the treatment of samples [69]. The acetyl groups are the unique non-sugar functional groups present in acemannan and seem to play an important role in the physical properties, biological activity, and topology of AV [440]. Acemannan presents good biodegradability and biocompatibility and has a wide range of applications in the biomedical field, due to its excellent immunomodulatory, antiviral, antitumor, bactericidal, antifungal, anticancer, and anti-inflammatory activities and tissue regeneration effects [441]. Its deacetylation affects its physical properties and bioactivity [440], and the degree of acetylation may differ in the commercial beverages containing AV, as reported in a recent study by Comas-Serra et al. (2023) [442] on 15 labelled beverages. It is crucial to consider the effects of the various drying procedures used to obtain acemannan from AV, since the modification of the acemannan structure may affect the beneficial properties of AV. Indeed, almost all drying methods may cause a considerable degradation of the acemannan structure, leading to a dramatic reduction in the functional properties [438].

## 7. Studies on Nanoparticles, Phytosomes, and Liposomes Containing *Aloe vera*

Nanoparticles containing metals and AV have been widely described for their biological activities. Silver NPs fabricated from AV extract showed antimicrobial [90,443,444,445,446], antioxidant [447], wound healing, antiparasitic [100,101], UV protecting [448], and catalytic activities [449]. Additionally, gold NPs evidenced antibacterial activity against *E. coli* and *S. aureus* [450], and selenium NPs synthesized from AV exerted antioxidant [451], antibacterial, and antifungal activities [452]. Titanium dioxide NPs obtained from AV have also been examined for their photocatalytic activity against methylene blue dye [453]. The use of AV extract enhanced the antimicrobial activity of biotemplated TiO_2_ nanoparticles [454]. Metal oxide NPs have also demonstrated interesting results; for example, CuO NPs showed antimicrobial activity [455] and the photocatalytic degradation of dye [456]. Ramiréz et al. (2024) [457] reported a study on nanovesicles (NVs) derived from AV peels (AVpNVs) as anti-inflammatory agents, which simultaneously diminished myofibroblast differentiation and contraction. The study was carried out in two macrophage cell lines stimulated with LPS (Raw264.7, THP-1 M0 phenotype), fibroblasts (HDNFs), and keratinocytes (HaCat) stimulated with LPS, via MTT and LDH assays. A strong anti-inflammatory effect was demonstrated in human macrophages, as well as human keratinocytes. AVpNVs also inhibited TGFβ-stimulated myofibroblast differentiation and reduced the contractile properties of fibroblasts and myofibroblasts in vitro. The study by Yahya et al. (2022) [83] demonstrated that the incorporation of AV gel with chitosan nanoparticles (CSNPs) enhanced the bacteriostatic activity of AV gel against *H. pylori*, as well as its antioxidant and hemolysis inhibition properties. Valizadeh et al. (2022) [458] reported an in vivo study of AV nanoemulsion gel containing erythromycin for topical antibacterial therapy in male Wistar rats. The group treated with AV nanoemulsion demonstrated a significant reduction in the epithelization period and wound contraction, compared with control groups, and a reduction in the number of inflammatory cells. Wu et al. (2017) [459] described a study on aloe-emodin nanoparticles compared to free AV in human lung squamous carcinoma. They demonstrated significant suppression of cancer cell proliferation in vitro, induced cell cycle arrest, and apoptosis through a high cleavage of caspase-3, poly (ADP-ribose) polymerase (PARP), caspase-8, and caspase-9. Nanoparticles also enhanced reactive oxygen species (ROS) production, along with mitogen-activated protein kinase (MAPK) activation and PI3K/AKT inactivation. Cell proliferation, apoptosis, and MAPKs and PI3K/AKT were dependent on ROS production in nano aloe-emodin-treated groups. In vivo, nanoparticles showed inhibitory effects on tumor growth, with low toxicity. Recent studies report the use of AV containing phytosomes and liposomes for diverse biological activities. Murugesan et al. (2021) [460] studied a phytosome gel prepared from the bio-encapsulation of AV extract with phospholipids, which demonstrated a high concentration-dependent inhibitory effect on MCF-7 cells in vitro. The use of phytosome carriers was suggested to enhance the oral delivery of AV, suggesting its use in cancer therapy. The encapsulation of AV extract into liposomes leads to a greater bioavailability and provides improved skin hypopigmenting activity, compared to the pure extract. In 2017, Ghafarzadeh & Eatemadi reported a double-blinded, randomized clinical study on the efficacy of a topical liposome encapsulating an AV leaf gel extract on pregnant women with melasma, a dermatological condition that occurs during pregnancy, obtaining better results compared to AV gel alone [461].

## 8. Metabolism of *Aloe vera*

AV is subjected to cytochrome P450 (CYP450) metabolism and significantly reduces the levels of CYP450 and cytochrome b5; thus, it is clearly an inducer of the phase-II enzyme system [462,463]. The work by Djuv & Nilsen (2012) [464] evidenced that two commercially available AV juice products exerted both CYP-mediated and non-CYP-mediated inhibitory activity towards recombinant human CYP3A4 and CYP2D6 enzymes and inhibited P-gp, in vitro. The study by Sen (2022) [465] demonstrated a weak inhibition of CYP1A1, CYP2B1/9, and CYP2E1 exerted by AV; moreover, AV likely blocks NAT enzymes, which are involved in the metabolism of mesalamine, one of the most widely prescribed medications for UC patients. Conversely, Brandin et al. (2007) [466] reported that AV juice promotes the expression of CYP1A2, CYP3A4, and P-gp in the human colon carcinoma cell line LS180. AV extract could be involved in the bioavailability of vitamins C and E, delaying their absorptive rate, probably through the formation of complexes with polyphenols [467]. Yang et al. (2017) [468] studied the interaction of AV with cyclosporine, an immunosuppressant with a narrow therapeutic window, which is a probe substrate of P-gp and CYP3A4. In vivo studies on male Sprague Dawley rats revealed that AV activated P-gp and AV metabolites derived from the activated CYP3A4. Most importantly, the systemic exposure to cyclosporine was significantly decreased by either a single dose or multiple doses of AV juice. However, studies on the single compounds revealed different activities, depending on the substrate used, in human liver microsomes. Liu et al. (2021) [469] found that emodin strongly inhibited the activities of human recombinant CYPs (CYP1A2, CYP2C9, CYP2C19), with IC_50_ values ranging between 0.67 and 7.62 μM and CYP1A2 and CYP3A4 (IC_50_ < 10 μM) in human liver microsomes. Interestingly, the CYP3A4-mediated metabolism of testosterone was strongly inhibited by emodin (IC_50_ = 9.6 μM), whereas for midazolam, only a weak inhibition was recorded (IC_50_ = 37.3 μM). CYP3A4 is the major metabolizing enzyme in both the liver and intestine; thus, its inhibition may lead to significant toxicokinetic interactions. Since emodin is commonly found in botanical laxatives, its intestinal concentration may reach adequate levels to inhibit CYP3A4 activity, with important consequences on the metabolism of drugs and other consumer products. Conversely, aloe-emodin inhibited CYP1A2, CYP2C9, and CYP2C19, with IC_50_ values under 10 μM [469]. The study by Hu et al. (2022) [470] showed that CYP3A4 and CYP3A1 are the main metabolizing enzymes of aloe-emodin in human and rat liver microsomes, respectively. Indeed, the authors suggest that the inhibition of CYP3A4 could enhance the hepatic damage caused by aloe-emodin. Particularly, aloe-emodin induces mitochondrial injury and the imbalance of oxidative stress in hepatocytes, triggering apoptosis. Meng et al. (2022) [321] reported that aloe-emodin acts as an inhibitor of CYP1B1 as well, a major estrogen-metabolizing enzyme that converts 17β-estradiol into 4-hydroxy-17β-estradiol, which may cause DNA damage and lead to tumor formation. Its inhibitory effect on CYP1B1 enzyme is higher than that of the isomer emodin, probably due to the position of the hydroxyl substituent The in vivo metabolism of aloin A, aloin B, and aloesin is still unclear. In a study by Kong et al. (2022) [471] in rats, twenty-five aloin A and B metabolites and three aloesin metabolites were found after the oral administration of aloin A, aloin B, and aloesin. The main biotransformation reactions were hydroxylation, oxidation, methylation, acetylation, and glucuronidation. Moreover, in vivo studies showed that aloin A and aloin B can be interconverted and that their metabolic profiles are identical. Niu et al. (2020) [472] reported that aloin A can rapidly pass into the blood circulation system, and is widely distributed in tissues. Finally, aloe-emodin may also be considered a metabolite, as it is the degradation product of aloin [341] and chrysophanol [473].

## 9. Valorization

The high demand for AV gel-based products, such as shampoo, soap, and sunscreen, has led to a surplus production of AV-processing waste. It should be considered that an AV gel processing facility may generate up to 4000 kg of AV waste per month. Nowadays, the AV waste is being disposed of in landfills or is used as fertilizer, but a sustainable management system for AV-processing waste should be envisaged. Several approaches have been proposed to valorize AV waste into value-added products, such as animal and 4aquaculture feeds, biosorbents, biofuel, and natural polymers [474]. The valorization of AV skin care by-products to obtain bioactive compounds has been recently investigated with interesting results. They were obtained by microwave-assisted extraction and showed antioxidant activity [475,476]. AV flowers, as well, are a by-product that could provide a valuable source of bioactive compounds with diverse functions for health benefits. The flowers of AV contain volatile compounds, amino acids, organic acids, sugars, trigonelline, fatty acids, phenols, carotenoids, and vitamin C. The composition depends on the maturity stage (immature; mature; mature, with flower buds opened). The study by Martínez-Sánchez et al. (2020) [477] evidenced a high antioxidant activity of AV flowers, containing significant vitamin C, total phenolic, and carotenoid contents, followed by citric and malic acids, trigonelline, and fatty acids, specifically PUFAs such as α-linolenic acid and linoleic acid, with an optimal balance between them. The major saturated fatty acids were found to be capric, palmitic, stearic, caprylic, and arachidic acids, whereas benzyl alcohol was one of the main volatile compounds. Recently, Ramírez et al. (2024) [461] reported that AV peel-derived nanovesicles show anti-inflammatory activity and prevent myofibroblast differentiation. Guancha-Chalapud et al. (2022) [478] studied the cellulose nanofibers obtained from AV cuticles, by using an acid hydrolysis method combined with ultrasound. Nanofibers were then added to acrylic hydrogels synthesized by the solution polymerization method. The acrylic hydrogels are very useful in agriculture to enhance the availability of water in soil, induce faster plant growth, increase plant survival with water stress, and allow the controlled release of fertilizers, therefore increasing crop yields. A recent study by Elefrjane et al. (2023) [479] addressed AV leaf waste extraction optimization for producing polyphenol-rich extracts to be used for the preparation of pharmaceutical and cosmetic formulations for the skin.

## 10. Toxicity Concerns of *Aloe vera*

Despite such widespread use of AV, several reports assessed its toxicity, specifically related to its activities in the liver, kidney and heart. Although a major part of the literature reports the protective effect exerted by AV against liver injury, several cases of acute liver injury have been also described [480,481]. For instance, Kanat et al. (2006) [482] reported the case of a 24-year-old man hospitalized with complaints of jaundice, pruritis, fatigue, and right upper abdominal discomfort, together with mild nausea and vomiting. He had no previous history of liver disease, blood transfusion, or alcohol consumption, but he took capsules containing 500 mg of AV extract for 3 weeks, before symptoms presentation. AV was immediately discontinued, and the patient’s symptoms resolved completely within 7 days. Rabe et al. (2005) [483] described a case of hepatitis in a 57-year-old female likely linked to the ingestion of AV. Also in this case, the patient’s hepatitis resolved completely after discontinuing the ingestion. Curciarello et al. (2008) [484] reported the case of a 26-year-old man who developed severe acute hepatitis after the consumption of an AV tea. The symptom onset was 2–4 weeks after the AV tea consumption, and the patient improved after the withdrawal of the AV tea. Yang et al. (2010) [485] described three cases of AV-induced toxic hepatitis for three women, aged between 55 and 62 years, who had taken AV preparations orally for months, then were admitted to the hospital for acute hepatitis. Upon discontinuation of the aloe preparations, their liver enzymes returned to normal levels. Bottenberg et al. (2007) [486] described a case study concerning a 73-year-old female admitted to the hospital for acute hepatitis due to the consumption of oral AV capsules for constipation. Upon discontinuation of the oral AV, liver markers of hepatotoxicity returned to normal levels. Parlati et al. (2017) [487] reported a case of acute liver injury in a 68-year-old female caused by the ingestion of AV. Again, upon discontinuation of the oral AV, liver function tests returned to normal levels. Lee et al. (2014) [488] described the case of a 21-year-old female patient admitted to the hospital with acute toxic hepatitis after taking an AV preparation for four weeks. It should be highlighted that patients taking prescription drugs that are potentially hepatotoxic, such as methotrexate or leflonomid, should be careful or totally avoid aloe-containing products [489]. Kiliś-Pstrusińska et al. (2021) [490] indicated that the ingestion of AV preparations may be associated with diarrhea, hypokalemia, and kidney failure. Moosa (2023) [491] reported the case of a 20-year-old black African male with acute liver and renal failure occurring four days after ingesting home-made AV tea. A case of AV-associated liver injury has been recently found in a 56-year-old woman: when AV was stopped, the patient’s liver function tests and clinical situation improved on follow-up [492]. It is also reported that AV may increase the chance of hypokalemia and cause digitalis toxicity and arrhythmia [493]. Kumar et al. (2010) [494] studied the effects of AV gel on the repolarization state of the myocardium, heart rate, QRS complex, and QT interval using electrocardiographs in albino rats, showing that low doses of AV did not produce any changes in either the depolarization or repolarization state of the myocardium, but in high doses of 300 mg/kg body weight, the effects were notable, thus suggesting the ability of AV gel to modulate the activity of K^+^ channels. The QT prolongation determined by AV has been recently reported by Villaescusa et al. (2023) [495], who recently discussed the adverse effects of AV administered in combination with prescribed cardiovascular drugs, such as antithrombotic agents and diuretics. A rise in bleeding and hypokalemia were observed.

Toxicity concerns are often related to the anthraquinones present in AV, and some toxicity studies on the single components have been described above. Anthraquinones belong to the larger family of compounds known as hydroxyanthracene derivatives (HADs), listed as prohibited substances in food supplements, and tolerated in amounts < 1 mg kg^−1^ in marketed products [496]. In 2017, the European Food Safety Authority (EFSA) declared that HADs should be “considered as genotoxic and carcinogenic unless there are specific data to the contrary,… and that there is a safety concern for extracts containing hydroxyanthracene derivatives although uncertainty persists” [331], based on the results that demonstrated the in vitro genotoxicity of aloe-emodin, emodin, and the structurally related substance danthron, and the in vivo genotoxicity of aloe-emodin. The European Commission reports that “the hydroxyanthracene derivatives aloe-emodin and emodin and structurally related substance danthron have been shown to be genotoxic in vitro. Aloe extracts have also been shown to be genotoxic in vitro, most likely due to hydroxyanthracene derivatives present in the extract. Furthermore, aloe-emodin was shown to be genotoxic in vivo. The whole leaf aloe extract and structural analogue danthron were shown to be carcinogenic” [497]. However, to date, the investigation of HAD toxicity was based on in vitro and in vivo studies conducted mainly on a single molecule (specifically emodin, aloe-emodin, and rhein), rather than on the whole plant extract. Tinti et al. (2023) [498] recently performed a phytochemical qualitative–quantitative characterization of different plant extracts (*Cassia angustifolia*, *Rhamnus frangula*, *Rhamnus purshiana*, *Rheum palmatum,* and *Rheum raponticum*) to assess the toxic events of HAD, used as a single molecule in comparison with the whole plant extracts. Cell viability was evaluated by the cytotoxicity assays on Caco-2 cell lines, and an innovative shotgun proteomics approach, coupled with bioinformatic analysis, was applied to profile the differential proteome changes in response to the adopted treatment. Generally, it was observed that whether or not the single compounds resulted in toxicity in the Caco-2 cells, no significant toxicity was observed with the plant extracts. Baldi et al. (2021) [499] reported that AV gel does not present toxic effects, unlike whole-leaf extracts or AV latex. Hayes et al. (2022) [500] demonstrated the absence of the genotoxicity of a mixture of aloins A and B, two of the main anthraquinones found in AV, and a commercial AV gel beverage.

## 11. Conclusions

AV has been known since ancient times for its numerous biological properties. It is a source of many chemical compounds, and contains around 75 biologically active compounds, i.e., vitamins, minerals, enzymes, sugars, phenolic compounds, sterols, and amino acids. Several studies are available in the literature dealing with the involvement of AV in human health, thanks to its numerous biological properties. AV is used for wound healing, curing burns, minimizing frostbite damage, preventing damage to the skin from X-rays, preventing cancer, raising high-density lipoproteins, decreasing blood sugar in diabetics, combating allergies, and stimulating the immune system, as well as for its antioxidant, anti-inflammatory, antimicrobial, antiparasitic, laxative, and antiviral activities. AV juice is also useful in the treatment of gastrointestinal problems, such as indigestion, heartburn, and IBS. The wide use of AV in the cosmetic industry is related to its beneficial effects on people’s aesthetic appearance (skin, hair, nails). Moreover, the use of AV is very widespread for avoiding the wastage of perishable foods, which represents an enormous challenge in the food sector and requires effective mitigation strategies. The biological activities of AV have been mostly related to the anthraquinones present in the latex and chromones and APs present in the inner leaf, especially acemannan. However, several studies also address some activities of other compounds, including feralolide. Recently, the valorization of AV by-products from different parts of the plant to obtain bioactive compounds has been investigated, leading to interesting results. The biological properties of AV are due to its constituents, but not all the activities have yet been attributed to one compound or the other. The active ingredients may act alone or in concert. Although the main properties are attributed to the anthraquinones, chromones, and APs, today it is believed that the effects should be attributed to the synergistic action of all compounds. Many studies are carried out on crude extracts, which are complex mixtures and often vary, due to different plant sources and different extraction procedures. Several attempts have been made to isolate single, biologically active components, to examine their effects and clarify their functional mechanisms. More definite studies are those conducted on the single constituents that are commercially available. Several concerns are related to the potential toxicity of AV. This is generally studied in relation to anthraquinones, belonging to the HADs, specifically emodin, aloe-emodin, and rhein. However, some studies assess the absence of toxicity for these compounds. The aim of this review was to summarize the recent literature data on the biological activities of AV and/or the single components, with them having been obtained from the plant or being commercially available, and gather information on the health benefits of AV and its products. Despite so many benefits, people should pay attention to the possible side effects of AV and its potential interactions with drugs. However, given the conflicting data and opinions regarding AV toxicity, it is necessary to continue research to definitely evaluate the influence of this plant and its products on human health.

## Figures and Tables

**Table 2:** In vitro antiviral activity studies on AV gel.

Activity			Ref.
Antiviral activity	HCMV in cell culture	Diminution of DNA viral replication and plaque formation in HCMV-infected cells	[109]
	Influenza A virus in MDCK cells	Reduction in influenza A virus replication and autophagy inhibition in MDCK cells. Reduced transcription and protein expression of M1, M2, and HA following AVE exposure, in a concentration-dependent manner.	[110]
	HSV-1 (Vero cells infected with HSV-1)	High reduction in PFU of HSV-1 with 5% concentration AV gel.	[112]
	H1N1 (female SPF BALB/c mice infected with PR8 (H1N1)	APS extracted from AV significantly ameliorated the clinical symptoms and the lung damage of female SPF BALB/c mice infected with PR8 (H1N1), and significantly reduced the virus loads and mortality.	[113]
	MNV1	AV reduced MNV1 infectivity both on food vegetable surfaces and in liquid media.	[114]

Abbreviations: HCMV: human cytomegalovirus; AVE: AV ethanolic extract; PFU: plaque-forming units; HSV-1: herpes simplex virus type 1; MNV1: murine norovirus 1; APS: AV polysaccharide.

**Table 4:** Studies of *Aloe vera* in gastrointestinal diseases.

Activity			Ref.
Gastroesophageal Reflux Disease (GERD)	Randomized clinical trial	AV syrup was safe and well tolerated and reduced the frequency of GERD symptoms	[206]
	Five clinical studies on patients suffering from GERD	10 mL AV gel syrup twice daily significantly eliminated GERD symptoms with no adverse effects, when compared with omeprazole or ranitidine	[207]
Irritable Bowel Syndrome (IBS)	A systematic review and meta-analysis of randomized controlled trials, non-randomized controlled trials, retrospective and prospective cohort studies, and controlled before-and-after studies	*Aloe*-containing preparations were more effective than placebo in improving symptoms among all IBS subtypes, such as diarrhea-predominant IBS (IBS-D) and mixed-pattern IBS (IBS-M))	[208]
Inflammatory Bowel Disease (IBD)	In vivo DSS-induced acute experimental colitis mouse model	AV-derived nanovesicles were able to attenuate inflammation and enhance tight junction proteins for acute colitis treatment	[212]
	In vivo DSS-induced acute experimental colitis mouse model	Glucomannan protected mice from DSS-induced colitis, maintained intestinal barrier integrity by mitigating anoikis mediated by the Nrf2/mitochondria axis and reduced ROS levels by activating the Nrf2/Gpx2 cascade	[213,214]
	Subacute ulcerative colitis in healthy male C57BL/6 mice	APS was responsible for the anticolitic action, by alleviating colonic inflammation	[215]
	In vivo DSS-induced experimental colitis in 7-week-old male BALB/c mice	AV synergistically acted with heat-killed *Lactobacillus plantarum* L.137 (HK L.137)	[216]
Ulcerative Colitis (UC)	Placebo-controlled, randomized, double-blind trial involving 44 UC patients, studying the effects of oral AV gel (200 mL/day) over a period of four weeks	UC patients receiving oral AV gel (200 mL/day) showed a significant reduction in clinical and histological disease activity scores (compared to placebo); no serious side effects were recorded	[217]
	In vivo study on acetic acid-induced UC in adult male Wistar rats	AV gel significantly improved the clinical activity index and inflammation	[218]
	In vivo studies on 70 Sprague Dawley male rats	AV extract exhibited a therapeutic effect in TNBS-induced colitis; the oral route was less effective than the local and rectal ones	[219]

Abbreviations: GERD: Gastroesophageal Reflux Disease; IBS: Irritable Bowel Syndrome; IBD: Inflammatory Bowel Disease; DSS: dextran sulfate sodium; Nrf2: nuclear factor erythroid 2-related factor 2; UC: Ulcerative Colitis; APS: AV polysaccharide; TNBS: 2,4,6-trinitrobenzene sulfonic acid.

**Table 5:** Chemical composition of *Aloe vera*.

Structure	Name(s)	IUPAC Names	Ref.
**ANTHRAQUINONES AND ANTHRONES**			
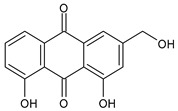	**Aloe-emodin**	1,8-Dihydroxy-3-hydroxymethyl-anthraquinone 3-Hydroxymethyl-chrysazin 1,8-Dihydroxy-3-hydroxymethyl-9,10-anthracenedione	[287]
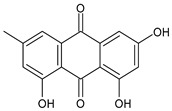	**Emodin**	1,3,8-Trihydroxy-6-methylanthraquinone 3-Hydroxy-6-methyl-chrysazin	[287]
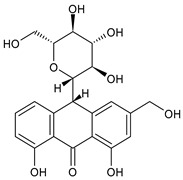	**Aloin A** **Barbaloin**	(10*S*)-Glucopyranosyl–1,8–dihydroxy–3–(hydroxymethyl)–9(10*H*)–anthraquinone 10-C-β-D-Glucopyranosyl-1,8-dihydroxy-3-(hydroxymethyl)-9(10*H*)-anthracenone	[287]
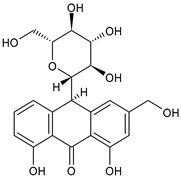	**Aloin B** **Isobarbaloin**	(10*R*)-10-Glucopyranosyl-1,8-dihydroxy-3-(hydroxymethyl)-9(10*H*)-anthraquinone (10*R*)-10-Glucopyranosyl-1,8-dihydroxy-3-(hydroxymethyl)-9(10*H*)-anthracenone	[287]
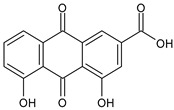	**Rhein** **Cassic acid** **Monorhein** **Rheic acid** **Rhubarb yellow**	4,5-Dihydroxy-9,10-dioxoanthracene-2-carboxylic acid 4,5-Dihydroxyanthraquinone-2-carboxylic acid 9,10-Dihydro-4,5-dihydroxy-9,10-dioxo-2-anthracenecarboxylic acid Chrysazin 3-carboxylic acid	[288]
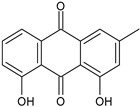	**Chrysophanol** **Chrysophanic acid**	1,8-Dihydroxy-3-methylanthraquinone-3-methylchrysazin	[131]
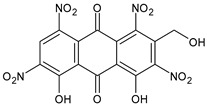	**Aloetic acid**	4,5-Dihydroxy-2-(hydroxymethyl)-1,3,6,8-tetranitroanthracene-9,10-dione	[64]
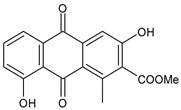	**Aloesaponarin I**	Methyl-3,8-dihydroxy-1-methyl-9,10-dioxoanthracene-2-carboxylate	[131]
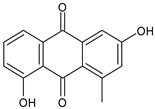	**Aloesaponarin II**	3,8-Dihydroxy-1-methylanthracene-9,10-dione 3,8-Dihydroxy-1-methyl-9,10-anthracenedione	[131]
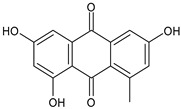	**Deoxyerythrolaccin**	1,3,6-Trihydroxy-8-methylanthracene-9,10-dione	[131]
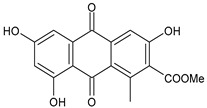	**Laccaic acid D-methylester**	3,6,8-Trihydroxy-1-methyl-9,10-dioxo-9,10-dihydroanthracene-2-carboxylic acid	[131]
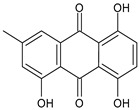	**Helminthosporin**	1,5,8-Trihydroxy-3-methylanthracene-9,10-dione	[131]
**GLYCOSIDES**			
**Glycosylated Chromones**			
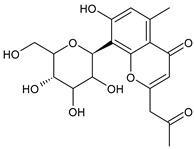	**Aloesin (formerly aloeresin B)**	–	[287]
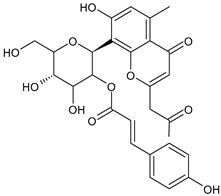	**Aloeresin A**	–	[287]
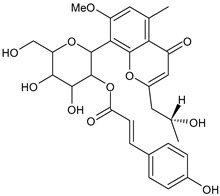	**Aloeresin D**	–	[287]
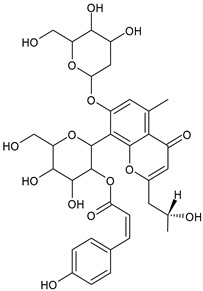	**Allo-aloeresin D**	–	[289]
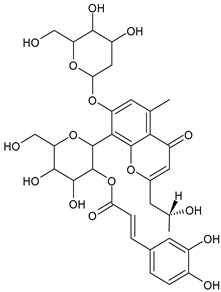	**Rebaichromone**	–	[289]
**Glycosylated pyran-2-ones**			
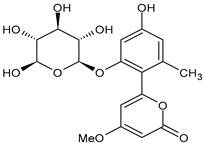	**Aloenin or Aloenin A**	–	[287]
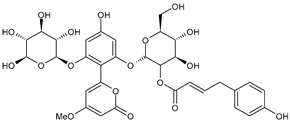	**Aloenin B**	–	[287]
**COUMARINS AND ISOCOUMARINS**			
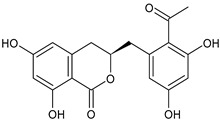	**Feralolide**	3*R*-3,4-dihydro-6,8-dihydroxy-3-(2′-acetyl-3′,5′-dihydroxyphenyl)- methylisocoumarin	[73]
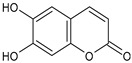	**Esculetin**	6,7-Dihydroxy-2*H*-1-benzopyran-2-one	[131]
**POLISACCHARIDES**			
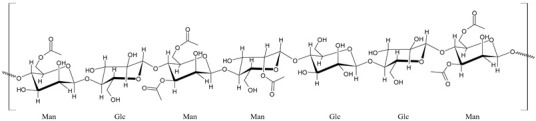 **Acemannan**			

## Data Availability

No new data were created or analyzed in this study. Data sharing is not applicable to this article.
